# Land use impacts on parasitic infection: a cross-sectional epidemiological study on the role of irrigated agriculture in *schistosome* infection in a dammed landscape

**DOI:** 10.1186/s40249-021-00816-5

**Published:** 2021-03-22

**Authors:** Andrea J. Lund, David H. Rehkopf, Susanne H. Sokolow, M. Moustapha Sam, Nicolas Jouanard, Anne-Marie Schacht, Simon Senghor, Assane Fall, Gilles Riveau, Giulio A. De Leo, David Lopez-Carr

**Affiliations:** 1 Emmett Interdisciplinary Program in Environment and Resources, Stanford University, 473 Via Ortega Suite 226, Stanford, CA USA; 2 Department of Epidemiology and Population Health, Stanford University School of Medicine, Stanford University, 1701 Page Mill Road Room 229, Palo Alto, CA USA; 3 Woods Institute for the Environment, Stanford University, 473 Via Ortega, Stanford, CA USA; 4 Hopkins Marine Station, Stanford University, 120 Ocean View Blvd, Pacific Grove, CA USA; 5 Centre de Recherche Biomédicale-Espoir Pour La Sante, 263 Route de la Corniche, BP 226, Saint-Louis, Sénégal; 6 Station d’Innovation Aquacole, UGB Cote Cite SAED, BP 524, Saint-Louis, Sénégal; 7 Center for Infection and Immunology of Lille, Institut Pasteur de Lille, 1 Rue du Professeur Calmette, 59800 Lille, France; 8 Department of Geography, University of California, 4836 Ellison Hall, Santa Barbara, CA USA

**Keywords:** Agriculture, Exposure, Livelihoods, Planetary health, Senegal, Schistosomiasis, Water contact

## Abstract

**Background:**

Water resources development promotes agricultural expansion and food security. But are these benefits offset by increased infectious disease risk? Dam construction on the Senegal River in 1986 was followed by agricultural expansion and increased transmission of human schistosomes. Yet the mechanisms linking these two processes at the individual and household levels remain unclear. We investigated the association between household land use and schistosome infection in children.

**Methods:**

We analyzed cross-sectional household survey data (*n* = 655) collected in 16 rural villages in August 2016  across demographic, socio-economic and land use dimensions, which were matched to *Schistosoma haematobium* (*n* = 1232) and *S. mansoni* (*n* = 1222) infection data collected from school-aged children. Mixed effects regression determined the relationship between irrigated area and schistosome infection presence and intensity.

**Results:**

Controlling for socio-economic and demographic risk factors, irrigated area cultivated by a household was associated with an increase in the presence of *S. haematobium* infection (odds ratio [*OR*] = 1.14; 95% confidence interval [95% *CI*]: 1.03–1.28) but not *S. mansoni* infection (*OR* = 1.02; 95% *CI*: 0.93–1.11). Associations between infection intensity and irrigated area were positive but imprecise (*S. haematobium:* rate ratio [*RR*] = 1.05; 95% *CI*: 0.98–1.13, *S. mansoni: RR* = 1.09; 95% *CI*: 0.89–1.32).

**Conclusions:**

Household engagement in irrigated agriculture increases individual risk of *S. haematobium* but not *S. mansoni* infection. Increased contact with irrigated landscapes likely drives exposure, with greater impacts on households relying on agricultural livelihoods.
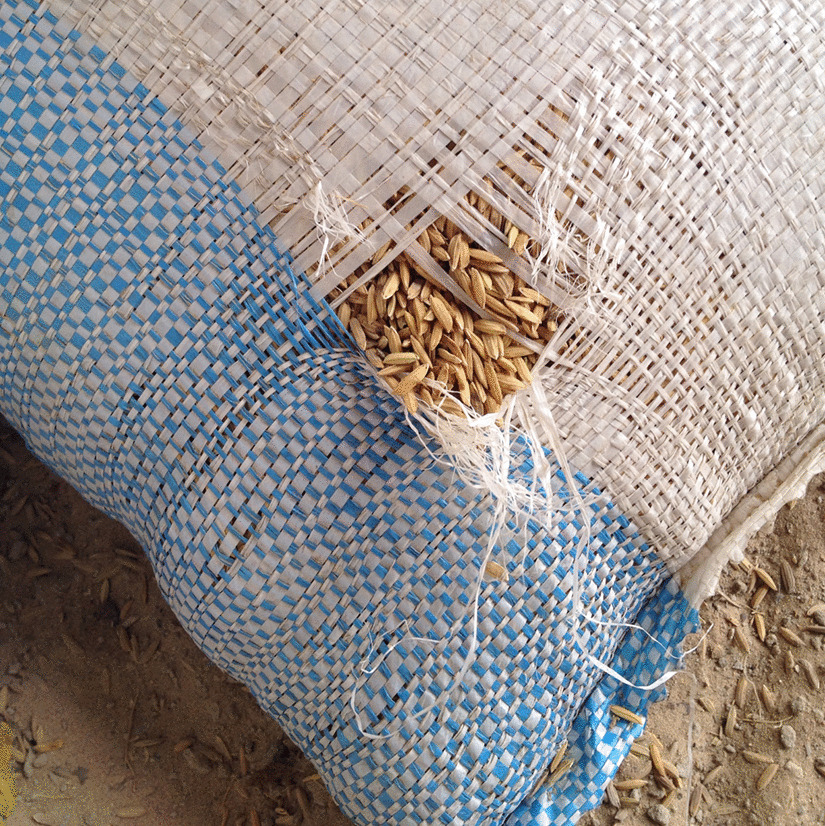

**Supplementary Information:**

The online version contains supplementary material available at 10.1186/s40249-021-00816-5.

## Background

Water resources development is increasingly important for meeting nutritional needs in low- and middle-income countries [1], but agricultural intensification is also a driver of some human infectious diseases [2, 3]. Exposure to pathogens transmitted through environmental pathways is often greater for those undertaking resource-dependent livelihoods. With few alternatives for reducing exposure, the resulting disease can impair economic development [4, 5]. As agriculture intensifies to meet human needs, it is important to understand unintended consequences of infrastructure designed to improve food security.

Irrigation infrastructure is often implicated in the increased occurrence of human schistosomiasis, a snail-borne parasitic disease second only to malaria in its global burden [6]. Parasitic worms of the genus *Schistosoma* inhabit the urogenital (*Schistosoma haematobium*) or the intestinal (*Schistosoma mansoni*) tract of the human host, with parasite eggs excreted in human urine and feces, respectively. Freshwater snails of the genera *Bulinus* serve as the intermediate host of *S. haematobium* while *Biomphalaria* transmit *S. mansoni*. These snail genera have distinct ecologies: *Bulinus* snails are able to withstand prolonged periods of drying while *Biomphalaria* are sensitive to saline conditions [7]. Species from both genera, however, can colonize irrigation canals [8–10]. Expansion of snail habitat and increased human activity in the aquatic environment both increase transmission in dammed and irrigated areas [11–13]. However, few studies disentangle the environmental and socio-behavioral mechanisms of schistosomiasis occurrence in areas where water resources are actively managed.

Of the approximately 800 million people at risk for schistosomiasis worldwide, 100 million live in close proximity to dams and irrigation schemes [6]. An estimated 200 million are infected, the majority of which live in sub-Saharan Africa [14, 15]. Parasites exiting snail hosts penetrate the skin of people who are in direct contact with water. Acute symptoms of schistosomiasis include hematuria for *S. haematobium* infection and diarrhea and abdominal pain for *S. mansoni* infection [16]. Prolonged infection can lead to anemia, organ damage, and cancer [17]. While lethal pathologies are linked to infection with both *S. haematobium* and *S. mansoni*, deaths are not often officially attributed to infection, and as a result, are likely underestimated [18]. Because available treatments do not prevent re-infection, regular contact with water can lead to chronic infection and severe disease. In some settings, prevalence and intensity of infection remain high even in the presence of treatment programs [19].

Existing evidence linking irrigated agriculture and schistosome infection relies on village- or landscape-level aggregations of disease occurrence and agricultural activity (Additional file 1), but finer-scale processes associated with irrigation influence exposure to parasites present in surface water. The household is a particularly relevant unit for both agricultural activity [20, 21] and water contact behavior [22, 23]. However, few studies investigate whether household-level circumstances compound infection risk present in the environment.

In this study, we investigated whether participation in agriculture at the household level was associated with individual-level schistosome infection. We assumed that in rural areas, the majority of schistosome exposure would occur at water access sites within a village and hypothesized that household-level cultivation of irrigated crops may represent additional exposure beyond the village-based exposure that occurs for most people. As a result, we suspected that children living households that cultivated irrigated land would be more likely to be infected with both species of schistosome. We further reasoned that, because larger areas of irrigated land are served by greater lengths of irrigation infrastructure, the occurrence of schistosomiasis would further increase in households that were cultivating larger areas of land. Larger fields served by more irrigation infrastructure would require more person-time to manage, harbor more snails, produce more parasites and, ultimately, increase schistosome exposure. With this rationale, we examined the relationship between area of irrigated land reported at the household level and individual-level infection outcomes, focusing on school-aged children who are often the target of mass drug administration campaigns.

While studies of both schistosomiasis ecology and water contact behavior often focus on the water access sites within a village, human contact with agricultural water sources could play an important role in sustaining schistosome transmission in a way that threatens the success of ongoing schistosomiasis control efforts. While the spatial distribution of agricultural water sources makes them difficult to monitor [23], processes of exposure and contamination in agricultural water sources may contribute to the contamination of village water sources and lead to re-infection after treatment [24, 25]. Such processes may contribute to the development of persistent hot spots of schistosomiasis transmission [26]. For schistosome transmission to be successfully suppressed, the implementation of interventions needs to account for the spatial scale of all the transmission sites in and around a village and the human movement between them.

The lower basin of the Senegal River became hyperendemic for schistosome transmission following the construction of the Diama dam in 1986 [11, 27], which was designed as a saltwater barrier to support agricultural development. Prior to dam construction, *S. haematobium* infections occurred seasonally at low levels, while *S. mansoni* was absent from the region [28]. By preventing saltwater intrusion, stabilizing water levels, promoting vegetation growth and disrupting the life histories of snail predators, the dam triggered environmental changes that favored the snail-borne transmission cycles of both *S. haematobium* and *S. mansoni* [29–31]*.* Salt-sensitive *Biomphalaria* have since become established in the perennially freshwater environment, while the irrigation infrastructure provides new habitat for both *Bulinus* and *Biomphalaria* [29, 30]*.* The prevalence and intensity of both infections remain high today in human populations, and agricultural practices have increasingly shifted to the cultivation of irrigated crops [32, 33]. Given the environmental changes affecting both parasite transmission and livelihoods in this setting, we aimed to understand whether household engagement in irrigated agriculture compounds the infection risk created by the dam, and whether agricultural sites of water contact may be involved in schistosome transmission in this setting. Specifically, we investigated whether schistosome infection increased in school-aged children living in households cultivating larger areas of irrigated land.

## Methods

### Study setting

**Fig. 1:**
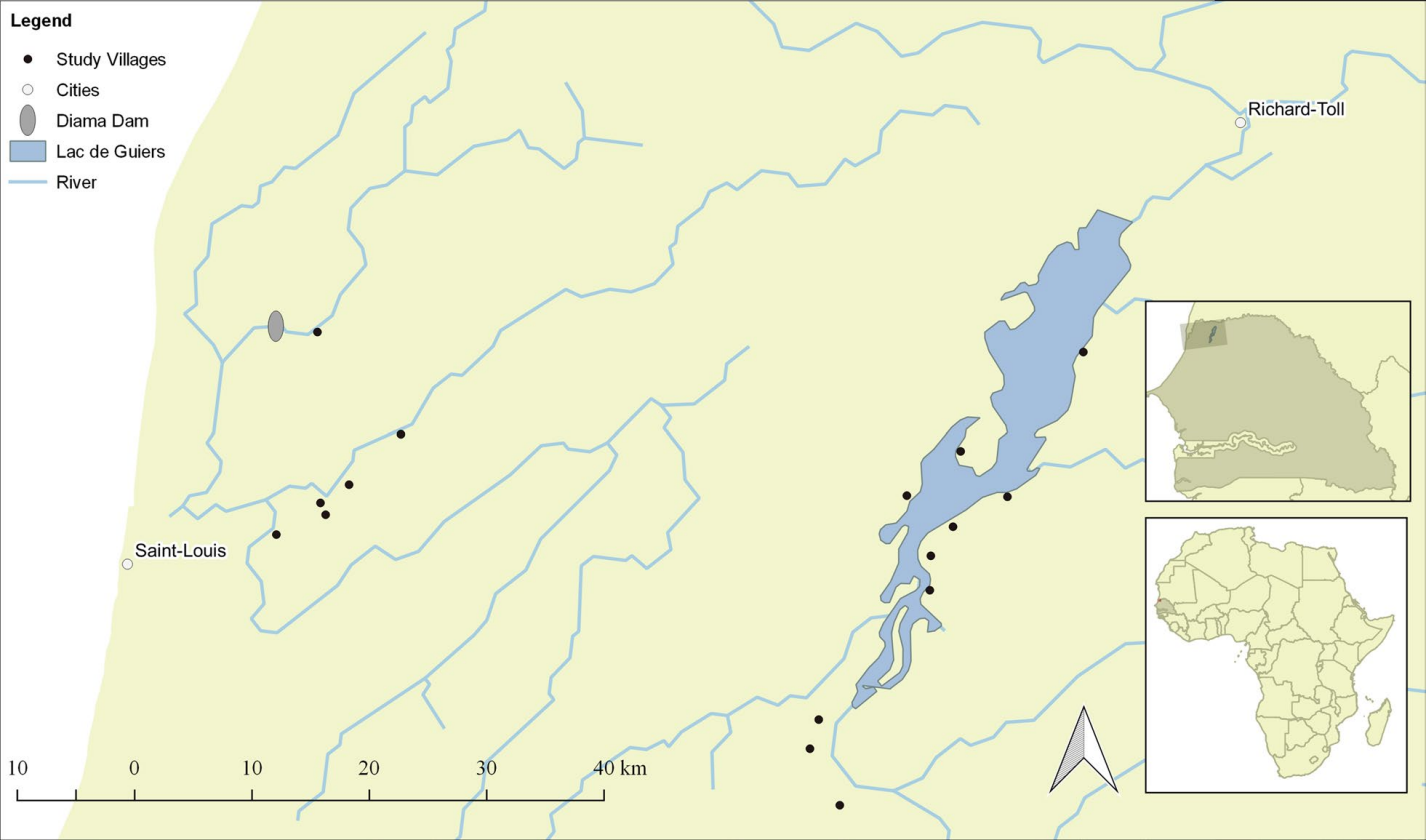
Study villages along the lower Senegal River along with freshwater bodies and the Diama Dam

This study used cross-sectional data collected as part of a longitudinal study of schistosome infection in school-aged children and the socio-economic conditions of the households where those children resided. Sixteen villages along the Senegal River, its tributaries and the Lac de Guiers in northwest Senegal were chosen to represent rural, high-transmission sites common in the region (Fig. 1). Village selection criteria are described in detail elsewhere [32], but included proximity to freshwater and presence of water access sites, presence of a school with sufficient enrollment in target grades and a non-zero prevalence of self-reported infection as well as accessibility in the rainy season. School-aged children were recruited from grades 1–3 in village schools. Agriculture was common in all villages with cultivation of irrigated rice using constructed irrigation infrastructure undertaken primarily in river villages and gardening and monocropping supported by hand-dug infrastructure in lake villages.

### Parasitological study procedures

**Fig. 2:**
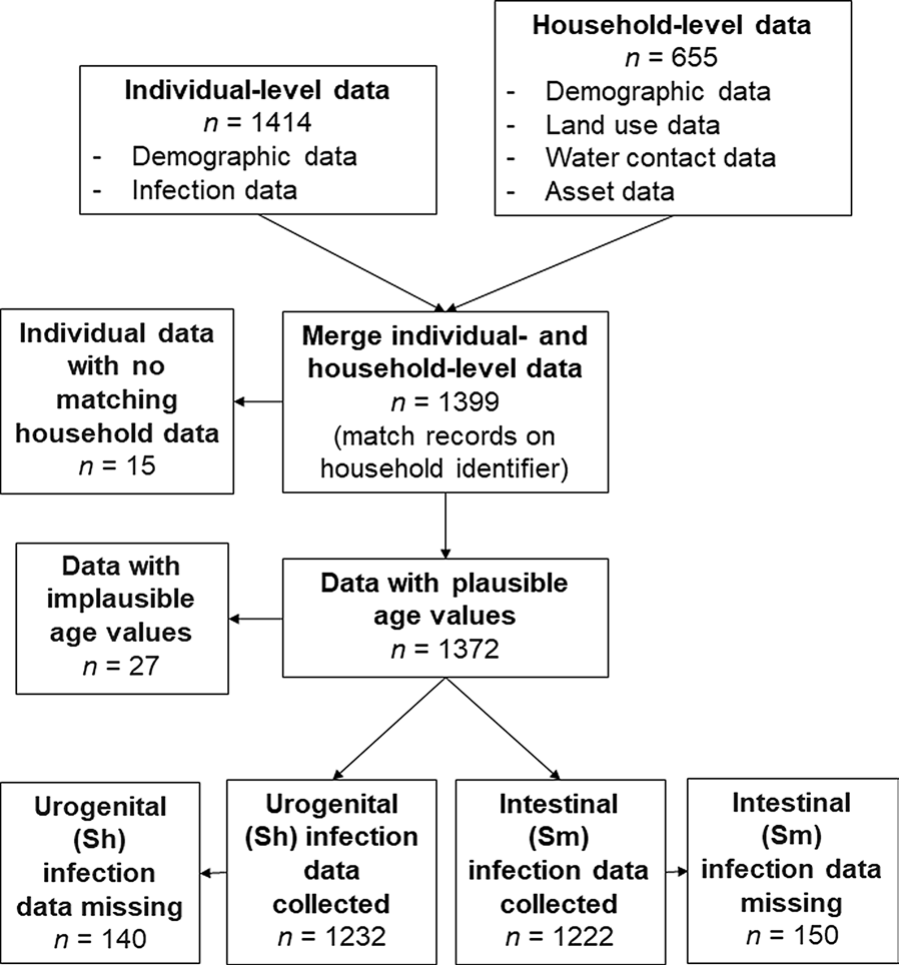
Assessment of sample size and missing data from combining household survey and parasitological data

Parasitological data were derived from a single year of a longitudinal, school-based parasitological study in all 16 villages (Fig. 1). A total of 1480 school-aged children were enrolled at baseline in February–April 2016. Of those, 1479 remained enrolled in January–April 2017 and 1414 successfully produced urine or stool samples on the two testing days that year (Fig. 2). On each testing day, one urine and one stool sample were collected from each child enrolled in the study. Urine and stool sample collection was organized at the school by trained personnel from the Biomedical Research Center Espoir Pour La Sante. Sampling pots were provided to each participant 24 h in advance, and samples were kept in isothermal boxes during transport (1–3 h) back to the laboratory. Samples were analyzed by urine filtration for *S. haematobium* infection and duplicate Kato-Katz examination of stool samples for *S. mansoni* infection by standard methods [34–36]. In each year of the longitudinal study, all children were treated with 40 mg/kg of praziquantel following sample collection. The cross-sectional parasitological data from 2017 data used in this study, thus, reflect post-treatment re-infection over the preceding year [37].

### Household survey data collection

Household survey were data collected in August 2016, during the rainy season preceding the 2017 parasitology data collection. We aimed to reach all the households where school-aged children enrolled in the parasitology study resided. The household survey instrument included six modules (Additional file 2). The modules used in this analysis included (1) demographic and (2) occupational information for every member of the household, (3) agricultural land use for all parcels owned and/or cultivated by household members, and (4) data on building materials and durable assets, which were used to approximate socio-economic status. Surveys were completed in 655 households (Fig. 2). The questionnaire was developed in English and translated to French by native speaking members of the field team. A team of eight Senegalese enumerators were trained to obtain verbal informed consent, pose survey questions in Wolof (the dominant local language) and record data in French. Prior to data collection, all survey questions were reviewed in French and the proper Wolof translations of key terms and ideas were discussed at length and agreed upon by all members of the enumerator team.

### Data processing and cleaning

Data from multiple urine (*n* = 2) and fecal (*n* = 4) observations for each child were summarized into single values of infection presence and intensity for each species of schistosome. Infections were determined to be present when any of the collected samples contained at least one schistosome egg. Infection intensity quantified egg counts (per 10 ml urine for *S. haematobium* and per gram feces for *S. mansoni*) by taking the median across the samples collected for each participant at both visits. Median values reduced the influence of missing or zero values.

Individual-level survey data were retained on the children for whom infection data were collected, while demographic data for remaining household members were aggregated to the household level (e.g. number of fishermen in the household, educational attainment of the household head). Parcel-level land-use data were also aggregated to the household level. Household-level asset data were used in a principal components analysis to generate an asset-based index of socio-economic status [38] (Additional file 3). We excluded individual observations from the merged data set that (1) had no matching record in the survey data, (2) reported ages outside the plausible rage (5–15 years) or (3) were missing outcome data for either *S. haematobium* or *S. mansoni* infection (Fig. 2).

### Statistical analysis

We used mixed effects regression to test the hypotheses that (1) the presence of *S. haematobium* and *S. mansoni* infections increased with irrigated land area (using logistic regression) and (2) the intensity of both infections (e.g. number of eggs detected in urine samples for *S. haematobium* and fecal samples for *S. mansoni*) increased with irrigated land area (using negative binomial regression). Mixed effects logistic regression was performed using the *lme4* package (version 1.1–21) [39] in R (version 1.1.456) [40], while mixed effects negative binomial regression was performed using the *glmmTMB* package (version 1.0.1) [41].

The primary model specification was determined from the hypothesized causal framework illustrated by a directed acyclic graph (DAG) (Additional file 4) [42]. In this specification, we controlled for prior common causes but not variables (1) on the hypothesized causal pathway between exposure and outcome nor (2) caused by exposure and outcome (i.e. colliders) to avoid introducing spurious non-causal associations and bias to the estimates of the relationship between exposure and outcome [43, 44]. With this model specification, we reduce bias that can occur by attenuating or inflating estimates [42]. However, to make this study comparable to prior models of schistosomiasis risk, we also present the results from an alternative model specification (Additional file 5) whose covariates were chosen based on model selection procedures outlined in an a priori analysis plan (Additional file 6).

For all models, we fit random intercepts of households nested within villages, using likelihood ratio (LR) tests to compare the fit of mixed effects models to those without random intercepts (Additional file 7). All statistical tests used an alpha level of 0.05. Because of perceived differences in the nature of agriculture performed in villages along the river and the lake, in all models, we tested two interaction terms between area of irrigated land and (1) the location of a village on the river or lake and (2) a household’s ownership of an irrigation pump, which allow for reduced exposure to surface water.

## Results

### Demographic characteristics of the study population

Of the 1372 school-aged children who provided urine and fecal samples in 2017 and whose households were successfully recruited for a household survey in 2016, the mean age was nine years [standard deviation (SD) = 2.0, varying slightly in lake (9.5 years) and river villages (8.4 years). The overall mean area of irrigated land (1.0 hectare) was similar across location strata (0.9 hectares on the river versus 1.0 hectares on the lake). A minority of households (19.4%) reported owning an irrigation pump, which was higher in lake (28.4%) than river villages (6.7%) (Table 1).

**Table 1:** Demographic characteristics of school-aged children and their households in 16 study villages, overall and by location

Variable	Level	Overall	River	Lake
Age [mean (SD)]		9.0 (2.0)	8.4 (1.6)	9.5 (2.1)
Sex [*n* (%)]	Female	665 (48.5)	274 (48.2)	391 (48.6)
Dominant ethnicity in household [*n* (%)]	Wolof	1021 (74.4)	265 (46.7)	756 (94.0)
	Pulaar	225 (16.4)	206 (36.3)	19 (2.4)
	Maure	87 (6.3)	68 (12.0)	19 (2.4)
	Other	39 (2.9)	29 (2.4)	10 (1.2)
Education of household head [*n* (%)]	None	966 (70.4)	274 (48.2)	692 (86.1)
	1–6 years	271 (19.8)	200 (35.2)	71 (8.8)
	7 + years	135 (9.8)	94 (16.5)	41 (5.1)
Number of wives taken by head [*n* (%)]	None	117 (8.5)	78 (13.7)	39 (4.9)
	One	809 (59.0)	382 (67.3)	427 (53.1)
	2 +	446 (32.5)	108 (19.0)	338 (42.0)
Irrigated area (hectares) [mean (SD)]		1.0 (2.2)	0.9 (2.8)	1.0 (1.7)
Pump ownership [*n* (%)]		266 (19.4)	38 (6.7)	228 (28.4)
Asset-based wealth quintile [*n* (%)]	1 (low)	210 (15.3)	89 (15.7)	121 (15.0)
	2	201 (14.7)	78 (13.7)	123 (15.3)
	3	240 (17.5)	91 (16.9)	149 (18.5)
	4	330 (24.1)	137 (24.1)	193 (24.0)
	5 (high)	391 (28.5)	173 (30.5)	218 (27.1)

SD Standard deviation

### Descriptive analysis of infection outcomes

Of the 1232 individuals with data on *S. haematobium*, the overall one-year prevalence of re-infection was 65.3% and varied from 43.1 to 82.1% across location strata. One-year re-infection prevalence for *S. mansoni* was 17.0% overall, with similar variation by location strata (Table 2). The geometric mean (GM) of *S. haematobium* infection intensity was 5.2 eggs per 10 ml urine (SD = 86.8) overall. Infection intensity was higher in lake villages (GM = 11.6 eggs per 10 ml, SD = 106.3) compared to river villages (GM = 1.4 eggs per 10 ml, SD = 39.8). The GM of *S. mansoni* infection intensity was 1.04 eggs per gram feces (SD = 215.7) (Table 2). We report GM to summarize extremely right-skewed egg count distributions for both infections [9] (Additional file 8).

**Table 2:** Prevalence and intensity of schistosome infection among school-aged children, overall and by location

Species	Measure	Overall	River	Lake
*Schistosoma haematobium*	Observations	1232	529	703
	Presence [*n* (%)]	805 (65.3)	228 (43.1)	577 (82.1)
	Intensity [GM (SD)]	5.2 (86.8)	1.4 (39.8)	11.6 (106.3)
*S. mansoni*	Observations	1222	527	695
	Presence [*n* (%)]	208 (17.0)	68 (12.9)	140 (20.1)
	Intensity [GM (SD)]	1.0 (215.7)	0.6 (87.7)	1.4 (274.8)

*GM* geometric mean, *SD* standard deviation

### Association between irrigated area on schistosome infection

**Fig. 3:**
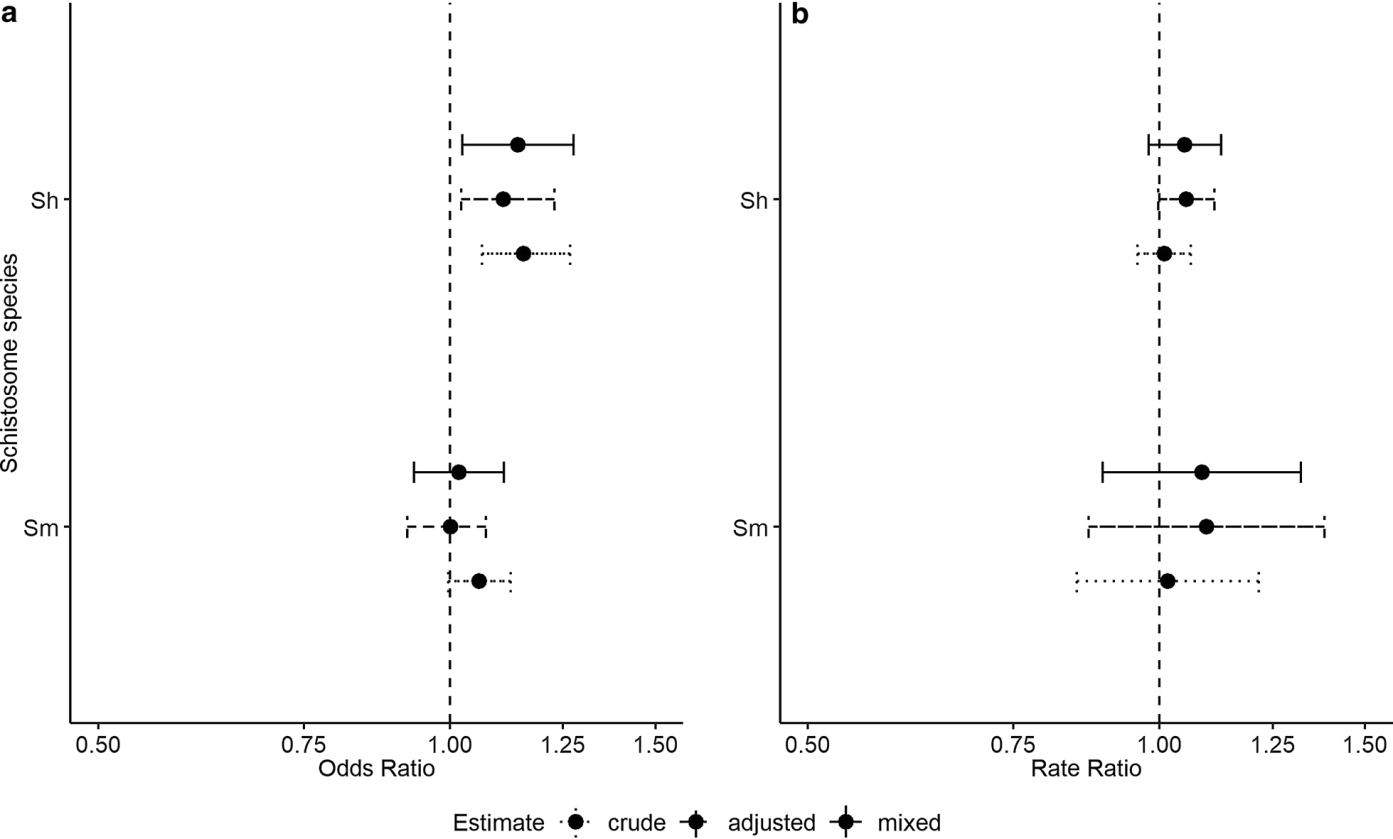
Estimates and 95% CIs for relationship between irrigated area and infection **a** presence and **b** intensity

For all four outcomes, we report estimates and confidence intervals for three distinct models: (1) crude bivariate models of exposure and outcome, (2) adjusted models that include DAG-based covariates and (3) mixed models that include DAG-based covariates and random intercepts accounting for the nested structure of the data (Fig. 3). In all cases, LR tests indicated the mixed models fit the data best compared to the crude and adjusted models (Additional file 7).

The odds of *S. haematobium* infection in children were higher with greater irrigated area (Fig. 3a, top). The point estimate indicates that the odds of infection in children increase 14% with every hectare increase in irrigated land reported by the household where a child lives. The 95% confidence interval (95% *CI*) for this estimate (1.03–1.28) is entirely above a value of no association (Table 3).

**Table 3:** Estimates and 95% confidence intervals (*CI*s) for the relationship between irrigated area and primary outcomes

Species	Infection presence		Infection intensity	
	Odds ratio	95% *CI*	Rate ratio	95% *CI*
*Schistosoma haematobium*	1.14	1.03–1.28	1.05	0.98–1.13
*S. mansoni*	1.02	0.93–1.11	1.09	0.89–1.32

For *S. mansoni* infection presence, the odds of infection did not increase with irrigated area. A small point estimate had a confidence interval that included a value of no association (OR: 1.02, 95% CI: 0.93–1.11; Fig. 3a, bottom, Table 3). The estimated 2% increase in the odds of *S. mansoni* infection presence with each hectare of irrigated land cannot be considered different from zero.

Similarly, intensity of infection did not increase with irrigated area. For both *S. haematobium* and *S. mansoni* infection intensity, point estimates from mixed effects negative binomial regression were greater than 1 (rate ratio (*RR*) = 1.05 for *S.haematobium*; *RR* = 1.09 for *S. mansoni*). However, both sets of confidence intervals included an *RR* of no association (95% *CI*: 0.98–1.13 for *S. haematobium*; 95% *CI*: 0.89–1.32 for *S. mansoni)* (Fig. 3b; Table 3).

Sensitivity analyses were largely supportive of the primary analyses (Additional file 9). While interactions between irrigated area and the two pre-specified variables (e.g., village location and pump ownership) improved the fit of crude or adjusted models, this was not true for any of the mixed models. For this reason, we report non-stratified estimates for each outcome (Fig. 3).

## Discussion

We find evidence that the occurrence of *S. haematobium* but not *S. mansoni* infections in a dammed landscape is compounded by engagement in agricultural livelihoods. In the lower basin of the Senegal River, the presence of *S. haematobium* infections in school-aged children increase with irrigated area cultivated by members of their households. This may result from greater contact with *Bulinus-* and *S. haematobium*-laden water among children whose families use and manage infrastructure for irrigating crops, compared to those whose contact occurs primarily at village water access sites. The observed association between irrigated area on *S. mansoni* infection presence was smaller and more uncertain, as were the associations of irrigated area with both measures of infection intensity, preventing firm conclusions about these outcomes. The contrast between *S. haematobium* and *S. mansoni* outcomes may reflect different sources of contamination in agricultural surface waters, such that the circulation of *S. haematobium* is more easily sustained by the input of urine than *S. mansoni* by the input of feces.

Our use of individual- and household-level data suggest that irrigated agriculture contributes to increased infection risk beyond the environmental consequences of infrastructure development. Previous meta-analysis on the topic revealed a greater increase in the occurrence of *S. mansoni* compared to *S. haematobium* in irrigated areas at the landscape scale [6]. This and other studies that have examined landscape-scale measures of disease occurrence and land use (Additional file 1) support the notion that human-mediated environmental change is associated with elevated infection prevalence, but do not shed light on the finer-scale mechanisms that influence individual infection risk. The relationship between disease risk and environmental exposures depends on the scales at which the relevant biotic, abiotic and human factors operate [45], such that individual- and landscape-level processes of disease and land use are not interchangeable and may represent distinct constructs [46]. In the lower basin of the Senegal River, dam construction in support of agricultural development has altered the landscape by stabilizing water levels, preventing saltwater intrusion and expanding the aquatic habitat available to the snails that transmit schistosomes [29]. Our use of finer-scale data establishes that processes related to household land use also play a role in determining risk for acquiring infection from the environment. The livelihoods made possible by the infrastructure contribute to infection risk in this landscape.

These findings also suggest that—beyond the in-village water access sites that are the typical focus of studies of schistosome ecology and water contact behavior—agricultural water sources play a role in sustaining schistosome transmission and connecting transmission sites to each other. The frequent use of irrigation canals for a wide variety of activities is likely to result in both snail-to-human and human-to-snail transmission in water sources outside a village [47]. This may be particular true for *S. haematobium,* whose eggs can be introduced more easily into the environment through urination compared to *S. mansoni*, whose eggs get introduced into the environment through defecation. If exposure and contamination occurs in both village and agricultural water sources, the human movement and water contact behaviors that connect these water sources will inevitably expand the spatial scope of transmission and the interventions needed to interrupt it [48]. Networks of water sources may ensure continuous introduction of parasites into a village, perpetuating transmission, threatening the success of both MDA and environmental interventions and potentially leading to the formation of persistent hot spots [26]. In this way, the design of interventions must account for the influence of human behavior on the ecological processes that affect infection risk at the proper scales.

As calls continue for environmental interventions to complement mass drug administration [49–51], the development of implementation guidelines should consider for the full spectrum of water contact activity and the disperse water sources that might contribute to transmission in a particular setting. The ability of water, sanitation and hygiene (WASH) interventions to reduce both exposure- and contamination-related behaviors, for example, may not be effective if agricultural water sources are disregarded [47, 52]. Environmental complements to MDA interventions may include cleaning aquatic vegetation to reduce snail habitat [53] and chemical and biological control of snail populations [51] in both water access points and irrigation canals.

This research has some limitations. Odds only approximate risk when outcomes are rare [54, 55]. Because the outcomes in this study are not rare, our estimates are biased away from the null compared to prevalence ratios. We attempted to directly estimate prevalence ratios by fitting log-binomial models [56], but these models did not converge. There are also limitations in the measurement of the variables we used for analysis. The exposure variable as well as covariates were all measured using self-reported survey data and subject to recall bias, which has been well described for exposure and disease studies [57]. We limited recall bias in the survey by anchoring the past in memorable events such as recent rainy and dry seasons as well as holidays. While survey respondents sometimes found it difficult to precisely quantify household land area, the evidence for recall bias in agricultural surveys in sub-Saharan Africa is limited [58].

Additionally, infection outcomes are limited by the sensitivity and specificity of available diagnostic methods: urine filtration for *S. haematobium* and duplicate Kato-Katz examination of two stool samples for *S. mansoni.* The detection methods used for *S. haematobium* are more sensitive compared to those used for *S. mansoni* [59, 60]*,* but the low sensitivity of diagnostic techniques used to detect *S. mansoni* infections—especially low intensity infections—may have contributed to the inconclusive results we observed for this parasite species [61].

Our findings add a new dimension to the notion that the benefits of water resources development for food security are offset by infectious disease. While we cannot speak to the dam’s net impact, we find that schistosomiasis risk may be a result of land use for subsistence livelihoods as well as landscape-level environmental change. Residents of the lower basin of the Senegal River face an unfortunate trade-off where the prevailing economic activity may make them sick.

## Conclusions

In finding elevated infection with *S. haematobium* among school-aged children living in houses cultivating larger areas of irrigated land, this study underscores the importance of designing and implementing schistosomiasis control interventions in ways that account for both human behavior and landscape complexity. The environmental changes resulting from water resources management affect how people interact with their environment, and help explain why high levels of infection persist in some endemic settings even in the presence of treatment programs. While our findings highlight the unfortunate complexity of persistent schistosomiasis transmission in the presence of mass drug administration, engaging the agricultural sector in environmental interventions to control transmission may be an important complement to drug-based strategies in settings where water resources management affects landscape-scale risk. Such complementary interventions may include environmental monitoring in agricultural landscapes, biological control of snails, infrastructure improvements in both the agricultural and health sectors and education of farmers whose families may be at a greater risk because of their livelihoods. Better informed interventions may help ensure that potentially deadly infection need not be the price paid for food security.

## Supplementary Information

**Additional file 1.** Existing literature on irrigated agriculture and schistosomiasis occurrence.

**Additional file 2.** Household survey instrument.

**Additional file 3.** Asset index construction.

**Additional file 4.**Directed acyclic graph (DAG) used to select covariates.

**Additional file 5.** Alternative model specification.

**Additional file 6.** Pre-specified analysis plan.

**Additional file 7.** Supplementary data for primary models.

**Additional file 8.** Distribution of egg counts.

**Additional file 9.** Sensitivity analysis.

**Additional file 10.** Supplementary data for alternative models.

## Data Availability

Village-level aggregations of the data used in this analysis have been deposited in the Dryad Digital Repository and can be found at: https://doi.org/10.5061/dryad.sf7m0cg4m. Individual- and household-level data needed to replicate the analysis while safeguarding anonymity can be made available by the corresponding author on reasonable request. All code used in the analysis is available online at https://github.com/andjanlund/schisto_irrigation.

## Abbreviations

DAG: Directed acyclic graph
GM: Geometric mean
*OR*: Odds ratio
*CI*: Confidential interval
*RR*: Rate ratio
SD: Standard deviation
*Sh*: *Schistosoma haematobium*
*Sm*: *Schistosoma mansoni*
