# Supplementary material for: Land use impacts on parasitic infection: a cross-sectional epidemiological study on the role of irrigated agriculture in schistosome infection in a dammed landscape
Source: Infect Dis Poverty. 2021 Mar 22;10:35. doi: 10.1186/s40249-021-00816-5 (PMC7983278; doi:10.1186/s40249-021-00816-5)
Supplement: Supplementary file 1 — Additional file 1. Existing literature on irrigated agriculture and schistosomiasis occurrence. [file 40249_2021_816_MOESM1_ESM.docx]

| **Table S1.** Summary of existing literature examining the relationship between agriculture and the occurrence of urogenital (Sh) and intestinal (Sm) schistosomiasis. | | | | | |
| --- | --- | --- | --- | --- | --- |
| **Setting** | **Agricultural exposure** | **Disease outcome** | **Sh** | **Sm** | **Ref** |
| Endemic countries | Living in an irrigated vs. non-irrigated area | Risk ratio | + | ++ | (1) |
| Mali | Intensively irrigated regions | Prevalence | + | + | (2) |
| Mali | Double- vs. single-crop rice cultivation | Prevalence | + | NA | (3) |
| Senegal River | Irrigated agriculture in village | Macro-haematuria | + | NA | (4) |
| Liberia | Swamp vs. upland rice farming | Prevalence | + | + | (5) |
| Cote d’Ivoire | Double- vs. single-crop rice cultivation | Prevalence | + | ++ | (6) |
| Egypt | Irrigated vs. rainfed cultivation | Prevalence | NA | + | (7) |
| Sierra Leone | Swamp rice development | Prevalence | NA | - | (8) |
| Sierra Leone | Swamp rice development | Prevalence | - | - | (9) |
| Ghana | Living in an irrigated area | Prevalence | + | NA | (10) |
| Egypt | Farmers vs. other occupations | Prevalence | NA | + | (11) |
| Mali | Living in a rice irrigated area | Co-infection | + | + | (12) |
| ++ indicates large positive relationship relative to other schistosome species, if applicable; + indicates positive relationship; - indicates no relationship; NA indicates relationship not examined | | | | | |

**References**

1. Steinmann P, Keiser J, Bos R, Tanner M, Utzinger J. Schistosomiasis and water resources development: systematic review, meta-analysis, and estimates of people at risk. The Lancet infectious diseases [Internet]. 2006;6(7):411–25. Available from: http://www.ncbi.nlm.nih.gov/pubmed/16790382

2. Brinkmann UK, Korte R, Schmidt-Ehry B. The distribution and spread of schistosomiasis in relation to water resources development in mali. Tropical Medicine & Parasitology [Internet]. 1988 [cited 2018 Sep 13];39:182–5. Available from: https://docxpress.stanford.edu/illiad.dll?Action=10&Form=75&Value=320456

3. Coulibaly G, Diallo M, Madsen H, Dabo A, Traoré M, Keita S. Comparison of schistosome transmission in a single- and a double-cropped area in the rice irrigation scheme, ‘Office du Niger’, Mali. Acta Tropica [Internet]. 2004;91(1):15–25. Available from: http://linkinghub.elsevier.com/retrieve/pii/S0001706X04000415

4. De Clercq D, Vercruysse J, Sene M, Seck I, Sall C, Ly A, et al. The effects of irrigated agriculture on the transmission of urinary schistosomiasis in the Middle and Upper Valleys of the Senegal River basin. Ann Trop Med Parasitol. 2000;94(6):581–90.

5. Kazura JW, Neill M, Peters PAS, Dennis E. Swamp Rice Farming: Possible Effects on Endemicity of Schistosomiasis Mansoni and Haematobia in a Population in Liberia. The American Journal of Tropical Medicine and Hygiene [Internet]. 1985 Jan 1 [cited 2020 Jan 29];34(1):107–11. Available from: http://www.ajtmh.org/content/journals/10.4269/ajtmh.1985.34.107

6. Yapi YG, Briët OJT, Diabate S, Vounatsou P, Akodo E, Tanner M, et al. Rice irrigation and schistosomiasis in savannah and forest areas of Côte d’Ivoire. Acta Tropica. 2005;93(2):201–11.

7. Hibbs AC, Secor WE, Gerven DV, Armelagos G. Irrigation and infection: The immunoepidemiology of schistosomiasis in ancient Nubia. American Journal of Physical Anthropology [Internet]. 2011 Jun 1 [cited 2020 Jan 29];145(2):290–8. Available from: https://onlinelibrary.wiley.com/doi/10.1002/ajpa.21493

8. White PT, Coleman M, Juppt BP. Swamp rice development, schistosomiasis and onchocerciasis in southeast Sierra Leone. American Journal of Tropical Medicine and Hygiene [Internet]. 1982 [cited 2018 Sep 10];31(3):490–8. Available from: https://www.publichealthjrnl.com/article/S0033-3506(05)80020-4/pdf

9. Gbakima AA. Inland Valley Swamp Rice Development: Malaria, Schistosomiasis, Onchocerciasis in South Central Sierra Leone. Public Health [Internet]. 1994 [cited 2018 Sep 10];108:149–57. Available from: https://www.publichealthjrnl.com/article/S0033-3506(05)80020-4/pdf

10. Bosompem KM, Bentum IA, Otchere J, Anyan WK, Brown CA, Osada Y, et al. Infant schistosomiasis in Ghana: a survey in an irrigation community. Tropical Medicine & International Health [Internet]. 2004 Aug 1 [cited 2020 Jan 29];9(8):917–22. Available from: https://onlinelibrary.wiley.com/doi/10.1111/j.1365-3156.2004.01282.x

11. Watts S, El Katsha S. Irrigation, farming and schistosomiasis: a case study in the Nile delta. International Journal of Environmental Health Research. 1997;7(2):101–13.

12. Mutombo N, Landouré A, Man WY, Fenwick A, Dembélé R, Sacko M, et al. The association between child Schistosoma spp. infections and morbidity in an irrigated rice region in Mali: A localized study. Acta Tropica [Internet]. 2019 Nov [cited 2020 Feb 10];199:105115. Available from: https://linkinghub.elsevier.com/retrieve/pii/S0001706X17314286
