# Supplementary material for: Land use impacts on parasitic infection: a cross-sectional epidemiological study on the role of irrigated agriculture in schistosome infection in a dammed landscape
Source: Infect Dis Poverty. 2021 Mar 22;10:35. doi: 10.1186/s40249-021-00816-5 (PMC7983278; doi:10.1186/s40249-021-00816-5)
Supplement: Supplementary file 2 — Additional file 2. Household survey instrument. [file 40249_2021_816_MOESM2_ESM.docx]

**Household survey instrument**

The development of the survey instrument was informed by previous surveys: one on migration and health designed by the senior author, Demographic and Health Survey questionnaires from Senegal and the 2012 Rural Agricultural Livelihoods Survey designed by researchers at the Indaba Agricultural Policy Research Institute in Lusaka, Zambia. The content of the household survey instrument used in this study, including the module, question and response categories is summarized in Table S2.

| **Table S2.** Household survey items used in in this analysis, including the module to which an item belonged, the level at which it was measured as well as the wording of and response categories provided for each question | | | |
| --- | --- | --- | --- |
| **Module** | **Level** | **Question** | **Response Categories** |
| Demographic characteristics | Individual | What is ____’s sex? | Male / Female |
|  | Individual | What is ____’s relationship to the household head? | Head / Spouse / In-law / Sibling / Child / Grandchild / Other relative / Non-relative |
|  | Individual | What is ____’s age? | [years] |
|  | Individual | What is ____’s ethnicity | Wolof / Pulaar / Maure / Serere / Other |
|  | Individual | Has ____ attended school? | Yes / No |
|  | Individual | What is the highest level of schooling ____ has completed? | [codes from 1-21 that correspond to grades in the French school system] |
| Occupational activity | Individual | What is ____’s primary occupational activity? | Cultivator / Gardener / Herder / Fisher / Trader / Artisan / Homemaker / Student / Retired / Day laborer / No activity / Other |
|  | Individual | Does ____ carry out additional occupational activities? | Yes / No |
|  | Individual | What is ____’s secondary activity? | Cultivator / Gardener / Herder / Fisher / Trader / Artisan / Homemaker / Student / Retired / Day laborer / No activity / Other |
| Agricultural land | Field | What is the area of the field? | [hectares] |
|  | Field | How is the field being used (cultivated)? | Sorghum / Millet / Onion / Rice / Peanut / Tomato / Fallow / Pasture / Wild / Other |
| Water contact | Site | Where is the site located? | In village / In field / Nearby village / Other |
|  | Site | Which members of the household visit the site? | Everyone / Men + women / Women + children / Men + children / Women only / Men only / Children only |
|  | Household | Do any of the following agricultural activities require contact with water? (Planting, plowing, weeding, irrigation, agrochemical treatment, harvesting) | Yes / No |
|  | Household | Which household members perform the following tasks? (Planting, plowing, weeding, irrigation, agrochemical treatment, harvesting) | Everyone / Men + women / Women + children / Men + children / Women only / Men only / Children only |
| Living conditions | Household | Is the household electrified? | Yes / No |
|  | Household | How many of [asset] does the household have? (assets include: radio, television, washing machine, gas stove, cooling fan, mobile phone, bicycle, cart, canoe, irrigation pump) | [number owned] |
|  | Household | How many rooms are in the household? | [number] |
|  | Household | What is the principal drinking water source in the household? | Piped water / Borehole / Well / Surface water / Other |
|  | Household | What is the principal water source used for washing clothes? | Piped water / Borehole / Well / Surface water / Other |
|  | Household | What type of toilet is primarily used by members of the household? | Flush toilet / Latrine / No toilet / Other |
|  | Household | What is the primary floor material? | Sand / Cement / Tile |
|  | Household | What is the primary roofing material? | Straw / Wood / Zinc / Cement / Shingles |
|  | Household | What is the primary material of the exterior walls? | Cement / Wood / Straw / Other |
