# Supplementary material for: Land use impacts on parasitic infection: a cross-sectional epidemiological study on the role of irrigated agriculture in schistosome infection in a dammed landscape
Source: Infect Dis Poverty. 2021 Mar 22;10:35. doi: 10.1186/s40249-021-00816-5 (PMC7983278; doi:10.1186/s40249-021-00816-5)
Supplement: Supplementary file 3 — Additional file 3. Asset index construction. [file 40249_2021_816_MOESM3_ESM.docx]

**Asset index construction**

Principal components analysis was used to generate an asset-based wealth index using the prcomp() function in the stats package (version 3.5.1) in R (1,2). Variables from the survey module on household conditions and durable assets were used, which ranged from the presence of electricity, to the quantity of mobile phones present in the home, to categorical variables of the household’s building materials. All categorical variables were converted to a single dichotomous variable for each category. Loadings for individual variables in the first principal component were used to calculate asset index for each household. Numeric asset scores were then divided into quintiles. The asset index used in the analysis was based on all 51 asset variables in the data. The first principal component explained 16% of the variation in the data.

Two additional principal components analyses were performed for subsets of the full set of 51 asset variables: (1) 20 variables comprising durable assets only and excluding building materials and water and sanitation infrastructure and (2) 40 variables for which standard deviation across the data set exceeded 0.2. The loadings on the first principal component did not differ across these three alternatives, nor did the distribution of numerical wealth scores or wealth quintile frequencies by village (Figure S1). We decided to retain the wealth index derived from the full set of asset variables.


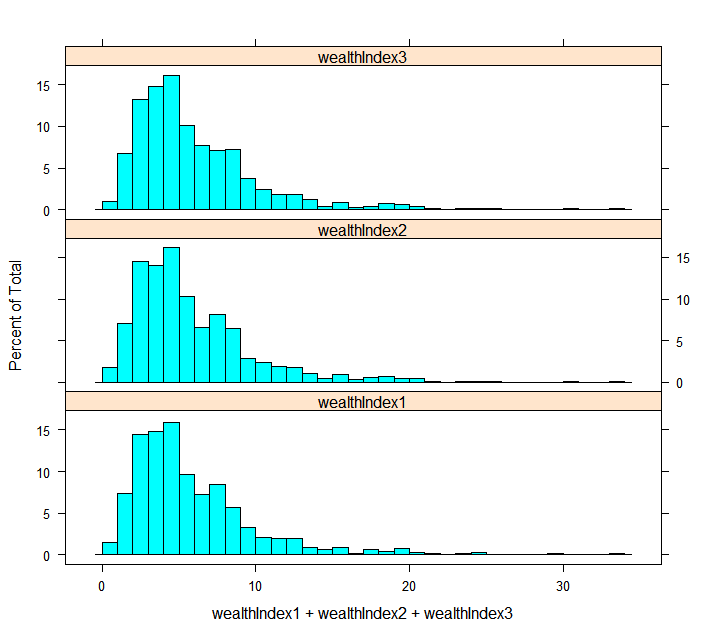


**Figure S1.** Distribution of wealth scores determined by the first principal component of principal components analysis on all asset data (wealthIndex1), durable asset variables only (wealthIndex2) and asset variables for which standard deviation exceeded a value of 0.2 (wealthIndex3).

**References**

1. Vyas S, Kumaranayake L. Constructing socio-economic status indices: how to use principal components analysis. Health Policy Plan [Internet]. 2006 Nov 1 [cited 2018 Nov 29];21(6):459–68. Available from: https://academic.oup.com/heapol/article/21/6/459/612115

2. Howe LD, Hargreaves JR, Huttly SR. Issues in the construction of wealth indices for the measurement of socio-economic position in low-income countries. Emerg Themes Epidemiol [Internet]. 2008 Jan 30 [cited 2018 Nov 29];5:3. Available from: https://www.ncbi.nlm.nih.gov/pmc/articles/PMC2248177/
