# Supplementary material for: Land use impacts on parasitic infection: a cross-sectional epidemiological study on the role of irrigated agriculture in schistosome infection in a dammed landscape
Source: Infect Dis Poverty. 2021 Mar 22;10:35. doi: 10.1186/s40249-021-00816-5 (PMC7983278; doi:10.1186/s40249-021-00816-5)
Supplement: Supplementary file 4 — Additional file 4.Directed acyclic graph (DAG) used to select covariates. [file 40249_2021_816_MOESM4_ESM.docx]

**Directed acyclic graph (DAG) used to select covariates**

Age and sex are well-known determinants of schistosome infection, independent of irrigated agriculture. The educational attainment of the household head and the number of wives reported by the household head are hypothesized to influence household wealth, which is approximated by an index of asset ownership. Asset ownership is hypothesized to influence how much irrigated land a household owns and cultivates, directly as well as through the ownership of an irrigation pump. We also hypothesized that the area of irrigated land in a household is influenced by two village-level variables: (1) the location of a village on the river or lake, capturing the ecological and other unmeasured differences between the two settings and (2) the amount of irrigated land in a given village (Figure S2).


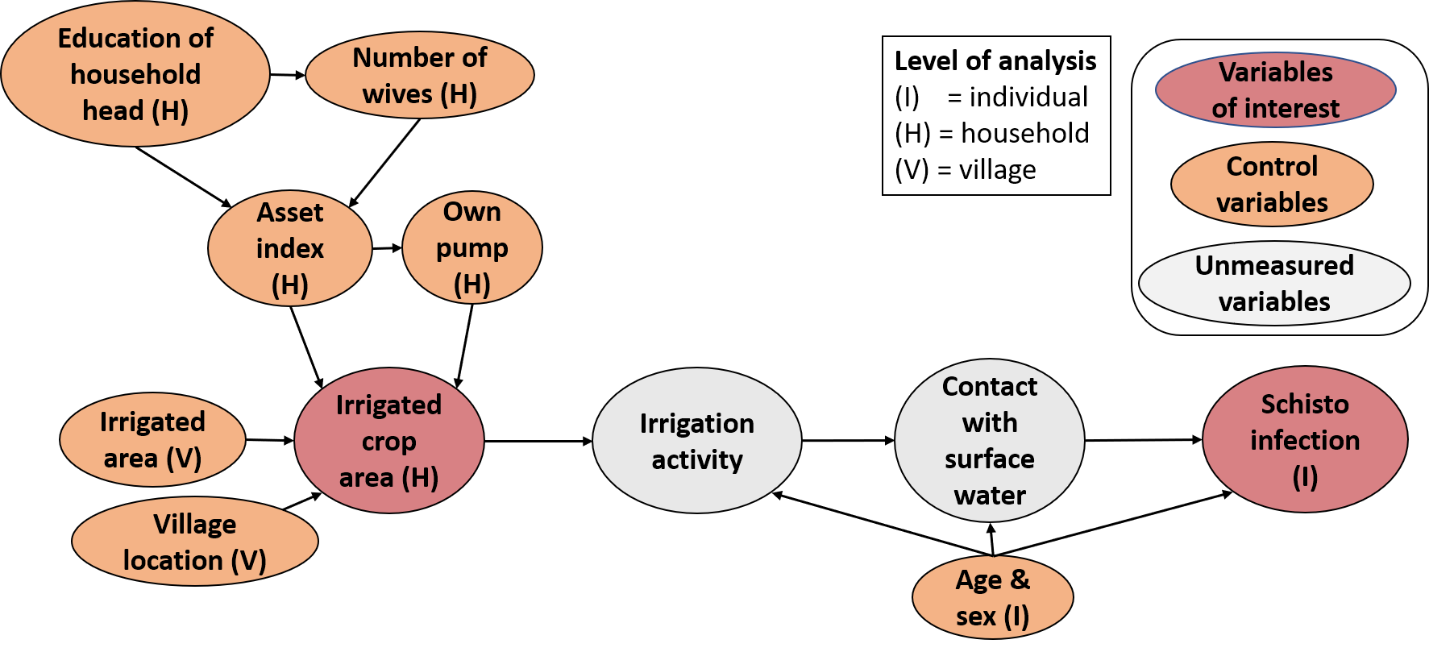


**Figure S2.** Directed acyclic graph illustrating the relationship between the two main variables of interest (irrigated crop area and schistosomiasis infection), their prior common causes and unmeasured intermediate variables in the causal pathway.
