# Supplementary material for: Land use impacts on parasitic infection: a cross-sectional epidemiological study on the role of irrigated agriculture in schistosome infection in a dammed landscape
Source: Infect Dis Poverty. 2021 Mar 22;10:35. doi: 10.1186/s40249-021-00816-5 (PMC7983278; doi:10.1186/s40249-021-00816-5)
Supplement: Supplementary file 5 — Additional file 5. Alternative model specification. [file 40249_2021_816_MOESM5_ESM.docx]

# **Alternative model specification**

In order to make this study comparable to prior models of schistosomiasis risk (1–6), we present the results from an alternative model specification whose covariates were chosen based on model selection procedures outlined in an *a priori* analysis plan. Correlations between primary outcomes and pre-specified covariates were used to screen variables for inclusion in regression models, using a significance threshold of p = 0.2. There was some heterogeneity in the directionality and significance of associations between covariates across the four primary outcome variables. When such heterogeneity occurred, we retained the covariate across all four models in order to maximize the comparability of coefficients across models (Table S3). Correlation coefficients between retained covariates were examined to assess potential for collinearity and variance inflation factors were inspected after model fitting.

For both binary and count outcomes, two interaction terms were tested between area of irrigated land reported by a household (irrigArea) and (1) the location of the household’s village on the river or the lake (loc) and (2) a household’s ownership of an irrigation pump (pump). These terms were added to full models containing all screened covariates. Nested models were compared using likelihood ratio tests (LRT). When models containing interaction terms improved the fit of the model (at a significance threshold of p < 0.1), we fit stratified models. We also included random effects for these models, using LRT to compare mixed effects models to those without random effects.

For all outcomes, estimates for crude (bivariate) models are shown as dotted lines (Figure S3). Models adjusted for all covariates are shown as dashed lines. Mixed effects models, which include all covariates plus random intercepts of households nested in villages are showed as solid lines. The model of Sm intensity (bottom right) was fit with village intercepts only, as the model could not be fit with nested intercepts (Figure S3). In all cases, the mixed effects model (solid line) represents the best-fitting model for that outcome.

| **Table S3.** Pre-specified covariates included for each primary outcome variable. Covariates were screened via bivariate correlation using a significance threshold of p < 0.20. Some covariates that were significantly correlated with one or more primary outcome variables and marginally significantly correlated with others were included for the sake of consistency in variable selection across models. | | | | | | |
| --- | --- | --- | --- | --- | --- | --- |
| **Variable** | **Description** | **level** | **Sh+** | **Sm+** | **nSh** | **nSm** |
| irrigArea | Irrigated area (hectares) | H | X | X | X | X |
| Sex (DEM01) | Child sex (1 = male; 2 = female) | I | X | X | X | X |
| Age (DEM04) | Child age (years) | I | X | X | X | X |
| riskActivity | Reported farming or fishing activity | I | - | - | - | - |
| Head_age | Age of household head | H | - | - | - | - |
| headEd | Education of household head (years) | H | X | X | X | X |
| nWives | Number of wives | H | X | - | X | - |
| eth_mode | Mode of ethnicities in household | H | X | X | X | X |
| nFishAny | Members with fishing activity | H | X | X | X | X |
| nWatPts | Total number of water points (WPs) | H | X | X | X | X |
| nWatPtsAgKids | Agricultural WPs used by children | H | X | X | X | X |
| kidsAnyTask | Children’s participation in agriculture | H | X | - | X | - |
| surface | Surface water used for domestic tasks | H | X | - | X | - |
| pump | Possession of water pump | H | X | X | X | X |
| hhsize | Size of household | H | - | - | - | - |
| wealth | Asset-based wealth quintile | H | X | X | X | X |
| distanceWatPt | Distance (meters) to nearest WP | H | X | X | X | X |
| distanceMarket | Distance to nearest market town | V | X | X | X | X |
| Loc | Location of village on river/lake | V | X | X | X | X |
| Sh+ = *S. haematobium* infection presence; Sm+ = *S. mansoni* infection presence  nSh = *S. haematobium* infection intensity; nSm = *S. mansoni* infection intensity  Level of analysis for individual (I), household (H) and village (V)  X indicates variable met correlation significance threshold for all or a logical subset of outcomes | | | | | | |


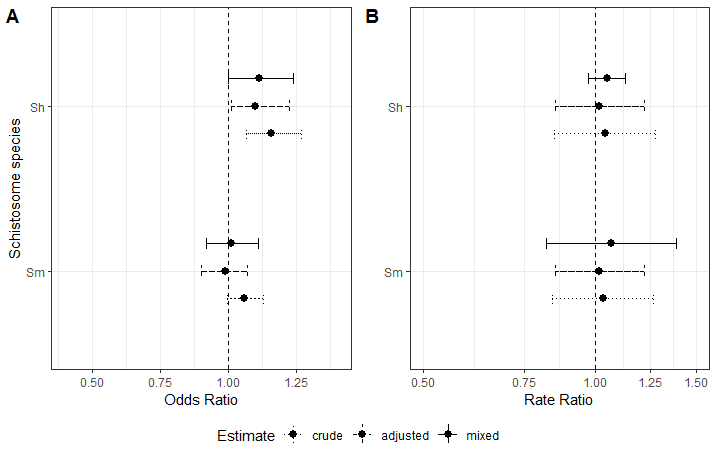


**Figure S3**. Estimates of irrigated area on schistosome infection presence and intensity using alternative (e.g. non-DAG-based) model specification. (A) Odds ratios with 95% confidence intervals drawn for mixed effects logistic regression models for *S. haematobium* (Sh, top) and *S. mansoni* (Sm, bottom) infection presence. (B) Rate ratios with 95% confidence intervals are drawn for negative binomial regression models of Sh and Sm infection intensity.

**References**

1. Chandiwana SK, Woolhouse ME. Heterogeneities in water contact patterns and the epidemiology of Schistosoma haematobium. Parasitology. 1991;103 Pt 3:363–70.

2. Kloos H, Fulford AJC, Butterworth AE, Sturrock RF, Ouma JH, Kariuki HC, et al. Spatial patterns of human water contact and Schistosoma mansoni transmission and infection in four rural areas in Machakos District, Kenya. Social Science & Medicine [Internet]. 1997 Apr [cited 2016 May 5];44(7):949–68. Available from: http://www.sciencedirect.com/science/article/pii/S0277953696002183

3. Steinmann P, Zhou X-N, Li Y-L, Li H-J, Chen S-R, Yang Z, et al. Helminth infections and risk factor analysis among residents in Eryuan county, Yunnan province, China. Acta Tropica. 2007;104:38–51.

4. Rudge JW, Stothard JR, Basáñez M-G, Mgeni AF, Khamis IS, Khamis AN, et al. Micro-epidemiology of urinary schistosomiasis in Zanzibar: Local risk factors associated with distribution of infections among schoolchildren and relevance for control. Acta tropica [Internet]. 2008 Jan [cited 2016 Apr 25];105(1):45–54. Available from: http://www.sciencedirect.com/science/article/pii/S0001706X0700229X

5. Pennance T, Person B, Muhsin MA, Khamis AN, Muhsin J, Khamis IS, et al. Urogenital schistosomiasis transmission on Unguja Island, Zanzibar: characterisation of persistent hot-spots. Parasites & Vectors [Internet]. 2016 [cited 2019 May 8];9. Available from: https://www.ncbi.nlm.nih.gov/pmc/articles/PMC5162088/

6. Wood CL, Sokolow SH, Jones IJ, Chamberline AJ, Lafferty KD, Kuris AM, et al. Precision mapping of snail habitats provides a powerful indicator of human schistosomiasis transmission. Proceedings of the National Academic of Sciences. 2019;116(46):23182–91.
