# Supplementary material for: Land use impacts on parasitic infection: a cross-sectional epidemiological study on the role of irrigated agriculture in schistosome infection in a dammed landscape
Source: Infect Dis Poverty. 2021 Mar 22;10:35. doi: 10.1186/s40249-021-00816-5 (PMC7983278; doi:10.1186/s40249-021-00816-5)
Supplement: Supplementary file 6 — Additional file 6. Pre-specified analysis plan. [file 40249_2021_816_MOESM6_ESM.docx]

**Pre-specified analysis plan**

In this appendix, we have included the analysis plan that was outlined prior to beginning analysis in May 2019. This plan includes hypotheses, procedures for covariate screening and model specification, testing interaction terms and fitting random effects as well as sensitivity analyses. The main diversion from this plan is the adoption of a DAG-based model specification, which constrained the number of covariates that were included in the model to prior common causes and excluded colliders and variables that were on the hypothesized causal pathway.

Above, we report the results from the pre-specified analysis plan—whose model specification is based on covariate screening using bivariate correlations. Since correlation-based model specifications are common for studies on risk factors of schistosomiasis (1–6), we report the results of this analysis for the sake of transparency and comparability with prevailing conventions in schistosomiasis epidemiology. The plan was outlined as follows:

*Objective and hypotheses*: The objective of this study is to understand how irrigated agriculture at the household level affects schistosome reinfection in school-aged children.

- H1: Presence and intensity of *S. haematobium* and *S. mansoni* infections will be higher in school-aged children who live in households cultivating larger areas.
- H2: In the subset of villages located on the river, prevalence and intensity of *S. haematobium* and *S. mansoni* will be higher in school-aged children who live in households cultivating larger areas of rice.

*Study design:* This study utilizes cross-sectional data from a longitudinal study of both schistosome infection in school-aged children and survey data from the households where those children reside.

*Village selection:* Sixteen villages along the Senegal River, its tributaries and the Lac de Guiers in northwest Senegal were chosen to be representative of the rural, high-transmission sites common in the region. Selected villages met the following inclusion criteria:

- Located in the immediate vicinity of the Diama Dam, on the Senegal River, Lac de Guiers or connected canals, with access to at least one regularly-used, permanent, freshwater access site
- Not in the top 10^th^ or bottom 10^th^ percentile of human population size, to obtain average size villages and ensure that selected communities could not be classified as urban
- Had a school with at least 30 students in grades 1-3 in order to enroll school-aged children in a longitudinal parasitological study
- Demonstrated non-zero schistosome infection rate, as determined by self-reported hematuria among school-aged children
- Were accessible by truck in the rainy season

*Participant selection:* School-aged children were recruited from grades 1-3 in the French school in each of the 16 villages. French schools provide secular education, which is compulsory in theory but not always enforced in areas where Islamic education is preferred. Treatment programs in the area are school-based and typically administered within French schools, making it possible that treatment programs fail to reach school-aged children who attend Coranic school but not French school.

A total of 1,480 school-aged children were enrolled at baseline in 2016 through the French schools in all 16 villages. Of those, 1479 remained enrolled during follow-up in 2017. In both years, outcome data are missing from some enrolled children, reflecting either absences from school on the day the parasitology team visited a given village and collected specimens or a child’s refusal or inability to produce a urine or stool sample during the team’s visit (Table S4). Missingness is higher during 2017 (Table S4). Urinary schistosome infection data is missing from 10% of enrolled children while intestinal schistosome infection data missing from 11% of enrolled children, compared to less than 1% of infection outcome data missing at baseline in 2016.

| **Table S4.** Children enrolled and outcomes observed at baseline and follow up for *S.haematobium* (Sh) infection and *S. mansoni* (Sm) | | |
| --- | --- | --- |
|  | **Number of observations**  **(% missing)** | |
|  | **Baseline - 2016** | **Follow-up - 2017** |
| Children enrolled in parasitology study | 1480 | 1479 |
| Children reached in 2016 household survey | 1429 | NA |
| Children in survey *and* parasitology datasets | 1416 | 1414 |
|  | (4.3) | (4.4) |
| *S. haematobium* infection presence | 1415 | 1271 |
|  | (4.4) | (10.1) |
| *S. haematobium* infection intensity | 1415 | 1266 |
|  | (4.4) | (10.5) |
| *S. mansoni* infection presence | 1404 | 1261 |
|  | (5.1) | (10.8) |
| *S. mansoni* infection intensity | 1404 | 1258 |
|  | (5.1) | (11.0) |
| *S. haematobium-S.mansoni* coinfection presence | 1404 | 1261 |
|  | (5.1) | (10.8) |

Household-level survey data were collected from all the households where children enrolled in the school-based study resided in all 16 villages. Additional missingness arises when merging parasitology and household survey data sets, as approximately 4% of the households where children enrolled in school-based parasitology study reside were not reached during the household survey.

*Outcomes:* As part of the longitudinal study of schistosomiasis in these villages, duplicate stool and urine samples were collected annually from enrolled school-children during in the spring months of 2016, 2017 and 2018. Urine filtration and Kato-Katz analyses determined the presence and intensity of infection at each time point. Following these analyses, each infected child was offered treatment of Praziquantel at 40 mg/kg. Thus, infection data from 2017 and 2018 reflect reinfection over the course of the preceding year. Reinfection data from 2017 will be used in this study. Egg counts for *S. haematobium* and *S. mansoni* were averaged for analysis.

*Primary outcomes*

- Presence/absence of *S. haematobium*
- Presence/absence of *S. mansoni* infection
- Intensity of *S. haematobium* infection (continuous; eggs per 10 mL urine)
- Intensity of *S. mansoni* infection (continuous; eggs per gram feces)

*Secondary outcomes*

- Intensity of *S. haematobium* infection (categorical)
- Intensity of *S. mansoni* infection (categorical)
- Presence/absence of *S. haematobium*-*S. mansoni* co-infection

| **Table S5.** Thresholds for categorical formulations of schistosome infection intensity determined by WHO guidelines (7) | | | | | |
| --- | --- | --- | --- | --- | --- |
| **Species** | **Units** | **None** | **Low** | **Moderate** | **High** |
| *S. haematobium* | Eggs/10 mL | 0 | 1-49 | NA | 50+ |
| *S. mansoni* | Eggs/gram | 0 | 1-99 | 100-399 | 400+ |

*Sample size*: 1414 kids in 664 households across 16 villages

*Comparisons to test hypotheses:* Estimate parameters for area of irrigated agriculture adjusted for pre-screened covariates

- For all villages, self-reported area of land under irrigated crops by household as a general test of the hypothesis.
- For river villages only, self-reported area of land under rice by household since rice cultivation is the most intense (and supported by major irrigation infrastructure projects made possible by the Diama dam) in villages along the Senegal River and its tributaries.

*Parameters:* For logistic regression models of schistosome infection presence/absence, prevalence differences and prevalence ratios will be estimated. For negative binomial regression models of infection intensity, relative reduction in intensity of infection will be estimated. In all cases, adjusted parameters will be estimated. Inclusion of control variables will be based on screening of covariates outlined below.

*Effect modification:* Factors potentially affecting the exposure of interest—area of irrigated agriculture—will be assessed on both additive and multiplicative scales:

- A binary village-level variable classifying the location of communities as proximate to either the (1) Senegal River and its tributaries or (2) Lac de Guiers were considered the primary potential effected modifier
- A binary household-level variable indicating possession of a water pump

If there is an interaction between exposure and a selected covariate (i.e. p < 0.1) results will be presented as stratified tables.

*Control variables:* Descriptive analyses on the following covariates will be considered for inclusion in regressions. Variables that are strongly associated with the outcome variables (p < 0.2) will be included. Variables with little variation in the population will be excluded to improve precision and reduce the standard errors.

- Covariates will be considered across three levels of analysis:
  - Individual-level covariates
    - Age
    - Sex
    - Any risky occupational activity (agriculture or fishing)
  - Household-level covariates
    - Age of household head
    - Education of household head
    - Modal ethnicity
    - Number of spouses
    - Number of fishermen in household
    - Dependence on surface water for domestic chores (drinking, laundry)
    - Number of water points used by members of household
    - Number of agricultural water points used by children in household
    - Children’s participation in agricultural tasks
    - Distance to nearest water access point
    - Pump ownership
    - Wealth quintile
    - Household size
  - Village-level covariates
    - River versus lake geography
    - Distance to nearest market town

*Random effects:* To account for the lack of independence between observations, the inclusion of random effects at each level of observation will be considered. After screening and finalizing control variables listed above, the inclusion of random effects will be considered. First, random intercepts at the individual level will be added to the model and compared to an identical model with no random effects using the Likelihood Ratio Test (LRT). If significant, the random effect will remain in the model and random intercepts will be tested at higher levels of analysis (household, then village) using the same statistical test.

*Regression diagnostics:* Check model and distributional assumptions*:* (1) Test linearity assumption for presence/absence models using visual assessment of scatter plots. Consider transforming variables if obviously not linear, (2) Assess dispersion parameter for count models by comparing Poisson and negative binomial regressions using a likelihood ratio test*.* Check for influential values using Cooks distance*.* Check for multicollinearity. If variance inflation factors (VIF) exceed 5-10, consider removing variables from the model.

*Sensitivity analysis*

- Different formulations of total irrigated land area:
  - Categorical formulation: no irrigated land, small field (0-1 ha), medium field (1-5 ha) or large field (5+ ha)
  - Binary formulation: Presence/absence of irrigated land
- Measures of specific subtypes of irrigated land
  - Rice fields
    - Continuous
    - Categorical (same cut points as for total irrigated land area)
    - Binary presence/absence
  - Other irrigated monocrops (i.e. rice, manioc, onion)
    - Continuous
    - Categorical (same cut points as for total irrigated land area)
    - Binary presence/absence
  - Shoreside gardens (i.e. maraichage)
    - Continuous
    - Categorical (same cut points as for total irrigated land area)
    - Binary presence/absence

**References**

1. Chandiwana SK, Woolhouse ME. Heterogeneities in water contact patterns and the epidemiology of Schistosoma haematobium. Parasitology. 1991;103 Pt 3:363–70.

2. Kloos H, Fulford AJC, Butterworth AE, Sturrock RF, Ouma JH, Kariuki HC, et al. Spatial patterns of human water contact and Schistosoma mansoni transmission and infection in four rural areas in Machakos District, Kenya. Social Science & Medicine [Internet]. 1997 Apr [cited 2016 May 5];44(7):949–68. Available from: http://www.sciencedirect.com/science/article/pii/S0277953696002183

3. Steinmann P, Zhou X-N, Li Y-L, Li H-J, Chen S-R, Yang Z, et al. Helminth infections and risk factor analysis among residents in Eryuan county, Yunnan province, China. Acta Tropica. 2007;104:38–51.

4. Rudge JW, Stothard JR, Basáñez M-G, Mgeni AF, Khamis IS, Khamis AN, et al. Micro-epidemiology of urinary schistosomiasis in Zanzibar: Local risk factors associated with distribution of infections among schoolchildren and relevance for control. Acta tropica [Internet]. 2008 Jan [cited 2016 Apr 25];105(1):45–54. Available from: http://www.sciencedirect.com/science/article/pii/S0001706X0700229X

5. Pennance T, Person B, Muhsin MA, Khamis AN, Muhsin J, Khamis IS, et al. Urogenital schistosomiasis transmission on Unguja Island, Zanzibar: characterisation of persistent hot-spots. Parasites & Vectors [Internet]. 2016 [cited 2019 May 8];9. Available from: https://www.ncbi.nlm.nih.gov/pmc/articles/PMC5162088/

6. Wood CL, Sokolow SH, Jones IJ, Chamberline AJ, Lafferty KD, Kuris AM, et al. Precision mapping of snail habitats provides a powerful indicator of human schistosomiasis transmission. Proceedings of the National Academic of Sciences. 2019;116(46):23182–91.

7. Montresor A, Crompton DWT, Hall A, Bundy D a. P, Savioli L, Unit WHOD of C of TDS and IP. Guidelines for the evaluation of soil-transmitted helminthiasis and schistosomiasis at community level : a guide for managers of control programmes. 1998 [cited 2020 Jan 27]; Available from: https://apps.who.int/iris/handle/10665/63821
