# Supplementary material for: Land use impacts on parasitic infection: a cross-sectional epidemiological study on the role of irrigated agriculture in schistosome infection in a dammed landscape
Source: Infect Dis Poverty. 2021 Mar 22;10:35. doi: 10.1186/s40249-021-00816-5 (PMC7983278; doi:10.1186/s40249-021-00816-5)
Supplement: Supplementary file 7 — Additional file 7. Supplementary data for primary models. [file 40249_2021_816_MOESM7_ESM.docx]

**Supplementary data for primary models**

**Table S11.** Likelihood ratio tests comparing crude, adjusted and mixed models of infection presence (logistic regression) and intensity (negative binomial regression) of *S. haematobium* and *S. mansoni*. Adjusted models were compared to crude models. Mixed models were compared to adjusted models.

| **Species** | **Measure** | **Model** | **Χ^2^** | **df** | **P-value** |
| --- | --- | --- | --- | --- | --- |
| *S. haematobium* | Presence | Crude | REF | REF | REF |
|  |  | Adjusted | 223.25 | 13 | < 0.001 |
|  |  | Mixed | 94.69 | 2 | < 0.001 |
| *S. mansoni* | Presence | Crude | REF | REF | REF |
|  |  | Adjusted | 56.04 | 13 | < 0.001 |
|  |  | Mixed | 97.67 | 2 | < 0.001 |
| *S. haematobium* | Intensity | Crude | REF | REF | REF |
|  |  | Adjusted | 142.79 | 13 | < 0.001 |
|  |  | Mixed | 156.92 | 2 | < 0.001 |
| *S. mansoni* | Intensity | Crude | REF | REF | REF |
|  |  | Adjusted | 32.31 | 13 | 0.002 |
|  |  | Mixed | 64.65 | 2 | < 0.001 |

**Table S12.** Regression output for crude, adjusted and mixed effects logistic regression models of *S. haematobium* (Sh_presence) and *S. mansoni* (Sm_presence) infection presence

|  | Dependent variable: | | | | | |
| --- | --- | --- | --- | --- | --- | --- |
|  |  | | | | | |
|  | Sh_presence | | | Sm_presence | | |
|  | logistic | | glmm | logistic | | glmm |
|  | (1) | (2) | (3) | (4) | (5) | (6) |
|  | | | | | | |
| Constant | 0.51^***^ | 2.29^***^ | 2.34^***^ | -1.64^***^ | -0.75^*^ | -1.09^*^ |
|  | (0.07) | (0.38) | (0.55) | (0.08) | (0.42) | (0.62) |
|  |  |  |  |  |  |  |
| Irrigated area | 0.15^***^ | 0.11^**^ | 0.13^**^ | 0.06^*^ | 0.002 | 0.02 |
|  | (0.04) | (0.05) | (0.06) | (0.03) | (0.04) | (0.05) |
| Location (river) |  | -1.89^***^ | -2.07^***^ |  | -0.52^***^ | -0.26 |
|  |  | (0.16) | (0.52) |  | (0.20) | (0.59) |
|  |  |  |  |  |  |  |
| Sex (female) |  | -0.39^***^ | -0.38^***^ |  | -0.22 | -0.25 |
|  |  | (0.13) | (0.14) |  | (0.16) | (0.17) |
|  |  |  |  |  |  |  |
| Age |  | 0.10 | 0.13 |  | -0.02 | 0.07 |
|  |  | (0.07) | (0.08) |  | (0.08) | (0.09) |
|  |  |  |  |  |  |  |
| Head, 1-7 yrs school |  | 0.50 | 0.07 |  | -16.15 | -16.74 |
|  |  | (0.38) | (0.43) |  | (569.62) | (362.04) |
|  |  |  |  |  |  |  |
| Head, >7 yrs school |  | 0.19 | -0.06 |  | -0.22 | -0.28 |
|  |  | (0.16) | (0.18) |  | (0.21) | (0.24) |
|  |  |  |  |  |  |  |
| Wives = 1 |  | -0.22 | -0.13 |  | -0.40 | -0.28 |
|  |  | (0.24) | (0.25) |  | (0.28) | (0.29) |
|  |  |  |  |  |  |  |
| Wives ≥ 2 |  | -0.32 | -0.20 |  | -0.19 | -0.08 |
|  |  | (0.27) | (0.28) |  | (0.30) | (0.32) |
|  |  |  |  |  |  |  |
| Pump ownership |  | 0.03 | -0.29 |  | -0.26 | -0.21 |
|  |  | (0.20) | (0.22) |  | (0.21) | (0.25) |
|  |  |  |  |  |  |  |
| Asset quintile 2 |  | -0.04 | 0.27 |  | -0.20 | -0.35 |
|  |  | (0.25) | (0.27) |  | (0.30) | (0.32) |
|  |  |  |  |  |  |  |
| Asset quintile 3 |  | -0.31 | -0.19 |  | -0.47 | -0.54^*^ |
|  |  | (0.24) | (0.26) |  | (0.29) | (0.32) |
|  |  |  |  |  |  |  |
| Asset quintile 4 |  | -0.19 | 0.23 |  | 0.02 | -0.19 |
|  |  | (0.23) | (0.25) |  | (0.26) | (0.29) |
|  |  |  |  |  |  |  |
| Asset quintile 5 |  | -0.24 | 0.14 |  | -0.29 | -0.37 |
|  |  | (0.23) | (0.26) |  | (0.27) | (0.31) |
|  |  |  |  |  |  |  |
| Village irrigated area |  | 0.001 | -0.001 |  | 0.003^***^ | 0.003 |
|  |  | (0.001) | (0.003) |  | (0.001) | (0.003) |
|  |  |  |  |  |  |  |
|  | | | | | | |
| Observations | 1,232 | 1,232 | 1,232 | 1,222 | 1,222 | 1,222 |
| Log Likelihood | -788.05 | -676.42 | -627.46 | -555.76 | -527.74 | -478.91 |
| Akaike Inf. Crit. | 1,580.10 | 1,382.85 | 1,288.92 | 1,115.52 | 1,085.48 | 991.81 |
|  | | | | | | |
| Note: | ^*^p^**^p^***^p<0.01 | | | | | |


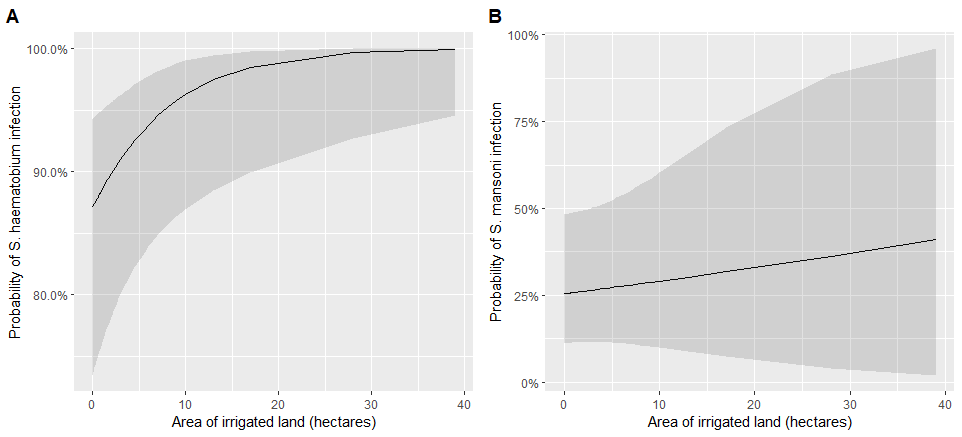


**Figure S5.** Prediction plots for DAG-based mixed effects logistic regression models of (A) probability of *S. haematobium* infection and (B) probability of *S. mansoni* infection across observed values of irrigated land area

**Regression diagnostics for mixed effects logistic regression models**

Regression diagnostics included checking model and distributional assumptions for logistic regression and negative binomial models. For count outcomes, calculation of the dispersion parameter and LRTs comparing nested Poisson and negative binomial models were used to discern which model best fit the data (1). We also checked for multicollinearity, removing variables from a model if variance inflation factors exceed a value of 5 (2). Cook’s distance was used to check for influential values in the model.


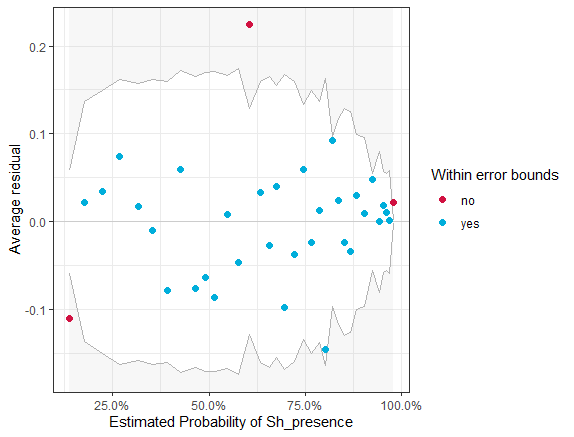

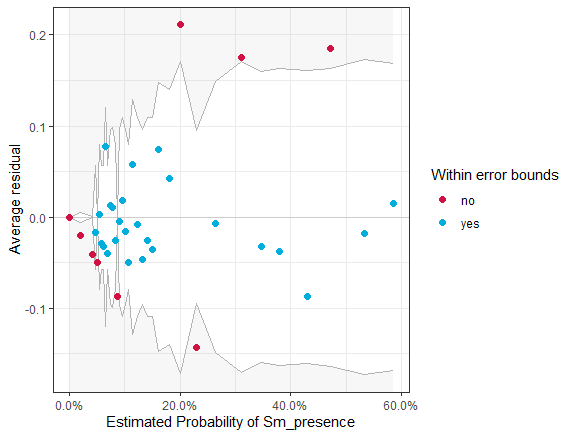


**Figure S6.** Binned residual plots for DAG-based mixed effects logistic regression models of schistosome infection presence. Left, 91% of binned residuals of *S. haematobium* presence fall within error bounds. Right, 74% of binned residuals of *S. mansoni* presence fall within error bounds. A model with 95% of the binned residuals within the error bounds is considered a very good fit, whereas a model with less than 80% of binned residuals within error bounds is considered a bad fit (3).


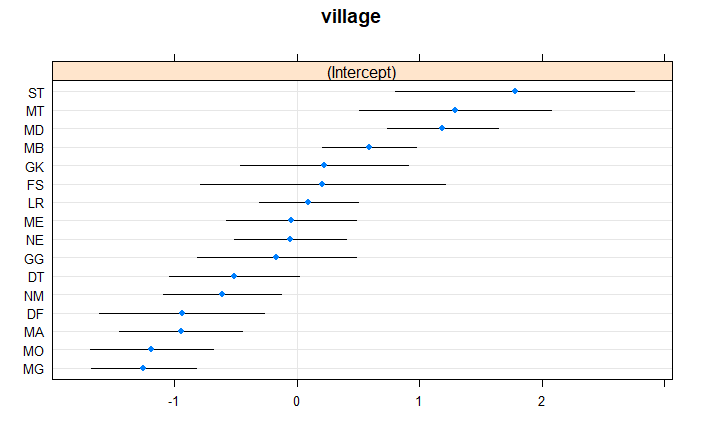

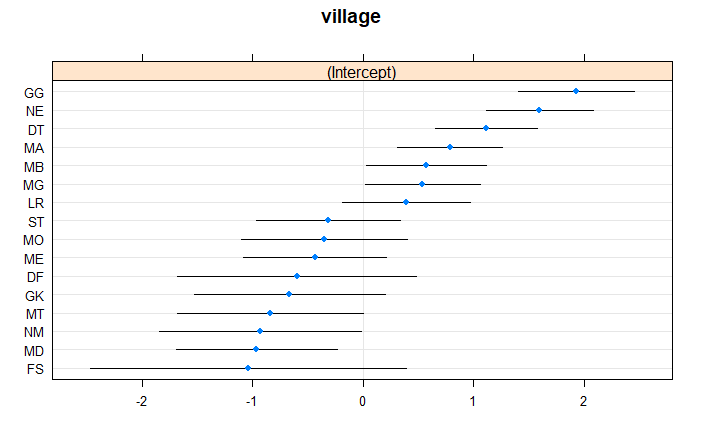


**Figure S7.** Point estimate and confidence intervals for village random intercepts in DAG-based mixed effects logistic regression models of *S. haematobium* presence (left) and *S. mansoni* presence (right)


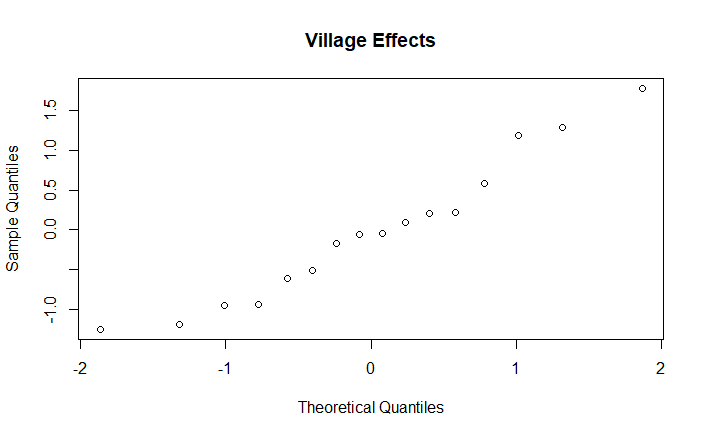

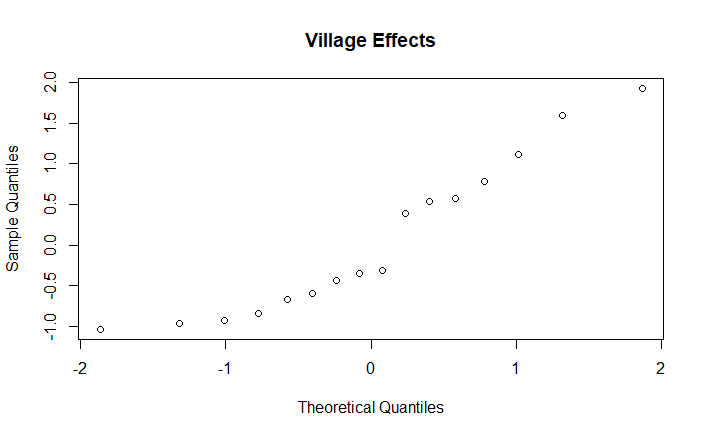


**Figure S8.** Q-Q plot of village random effects for DAG-based mixed effects logistic regression models of *S. haematobium* presence (left) and *S. mansoni* presence (right)

**Table S13.** Regression output for crude, adjusted and mixed effects negative binomial models of both *S. haematobium* (Sh_median) and *S. mansoni* (Sm_median) infection intensity

|  | Dependent variable: | | | | | |
| --- | --- | --- | --- | --- | --- | --- |
|  |  | | | | | |
|  | Sh_median | | | Sm_median | | |
|  | (1) | (2) | (3) | (4) | (5) | (6) |
|  | | | | | | |
| Constant | 3.55^***^ | 4.29^***^ | 3.87 ^***^ | 3.49^***^ | 4.64^***^ | 5.76^***^ |
|  | (0.07) | (0.38) | (0.59) | (0.22) | (1.10) | (1.71) |
|  |  |  |  |  |  |  |
| Irrigated area | 0.01 | 0.05^*^ | 0.05 | 0.02 | 0.09 | 0.08 |
|  | (0.03) | (0.03) | (0.04) | (0.09) | (0.12) | (0.10) |
| Location (river) |  | -1.59^***^ | -2.37^***^ |  | -1.32^**^ | -0.61 |
|  |  | (0.18) | (0.57) |  | (0.61) | (1.48) |
|  |  |  |  |  |  |  |
| Sex (female) |  | -0.04^***^ | -0.20 |  | -0.46 | -1.38*** |
|  |  | (0.14) | (0.14) |  | (0.49) | (0.49) |
|  |  |  |  |  |  |  |
| Age |  | -0.28 | -0.22** |  | 0.05 | 0.33 |
|  |  | (0.07) | (0.08) |  | (0.21) | (0.21) |
|  |  |  |  |  |  |  |
| Head, 1-7 yrs school |  | 0.41** | 0.10 |  | -28.10 | -24.54 |
|  |  | (0.36) | (0.45) |  | (29691.40) | (2561.48) |
|  |  |  |  |  |  |  |
| Head, >7 yrs school |  | -0.48** | -0.39 |  | -0.25 | -1.92*** |
|  |  | (0.16) | (0.23) |  | (0.61) | (0.72) |
|  |  |  |  |  |  |  |
| Wives = 1 |  | -0.13 | -0.27 |  | -0.04 | 0.01 |
|  |  | (0.25) | (0.31) |  | (0.73) | (0.74) |
|  |  |  |  |  |  |  |
| Wives ≥ 2 |  | -0.15 | -0.16 |  | 0.59 | -0.81 |
|  |  | (0.29) | (0.35) |  | (0.91) | (0.91) |
|  |  |  |  |  |  |  |
| Pump ownership |  | 0.12 | -0.11 |  | -0.74 | -1.23 |
|  |  | (0.18) | (0.24) |  | (0.61) | (0.64) |
|  |  |  |  |  |  |  |
| Asset quintile 2 |  | -0.24 | -0.14 |  | -0.40 | -0.64 |
|  |  | (0.24) | (0.30) |  | (0.78) | (0.94) |
|  |  |  |  |  |  |  |
| Asset quintile 3 |  | -0.17 | -0.26 |  | 0.04 | -2.47^***^ |
|  |  | (0.24) | (0.29) |  | (0.78) | (0.83) |
|  |  |  |  |  |  |  |
| Asset quintile 4 |  | -0.34 | 0.10 |  | 0.60 | -0.79 |
|  |  | (0.22) | (0.29) |  | (0.73) | (0.84) |
|  |  |  |  |  |  |  |
| Asset quintile 5 |  | -0.52* | -0.21 |  | -0.67 | -1.38 |
|  |  | (0.23) | (0.31) |  | (0.83) | (0.82) |
|  |  |  |  |  |  |  |
| Village irrigated area |  | 0.00 | 0.00 |  | -0.00 | 0.00 |
|  |  | (0.00) | (0.00) |  | (0.00) | (0.01) |
|  |  |  |  |  |  |  |
|  | | | | | | |
| Observations | 1,232 | 1,232 | 1,232 | 1,222 | 1,222 | 1,222 |
| Log Likelihood | -4,320.36 | -4,248.96 | -4,170.50 | -1,625.81 | -1,609.65 | -1,577.33 |
| Akaike Inf. Crit. | 8,646.71 | 8,529.92 | 8,377.00 | 3,257.62 | 3,251.31 | 3,188.66 |
|  | | | | | | |
| Note: | ^*^p^**^p^***^p<0.01 | | | | | |


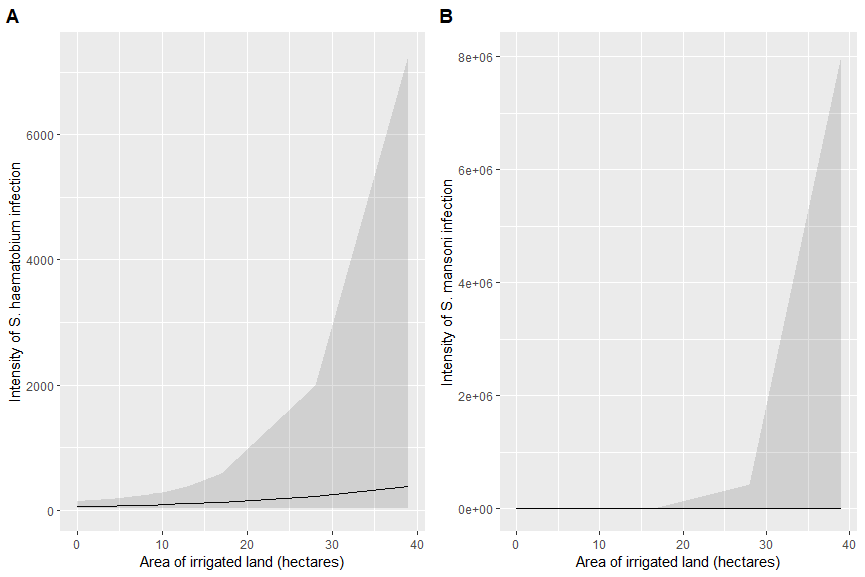


**Figure S9.** Prediction plots for DAG-based mixed effects negative binomial models of (A) intensity of *S. haematobium* infection and (B) intensity of *S. mansoni* infection across observed values of irrigated land area


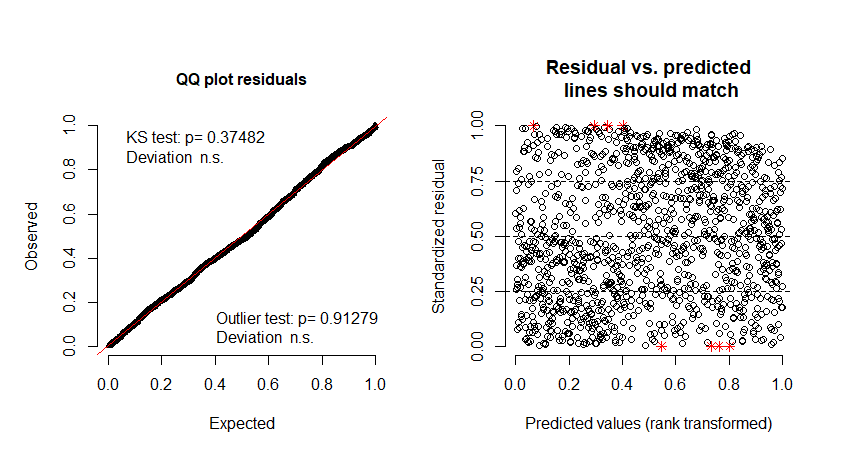


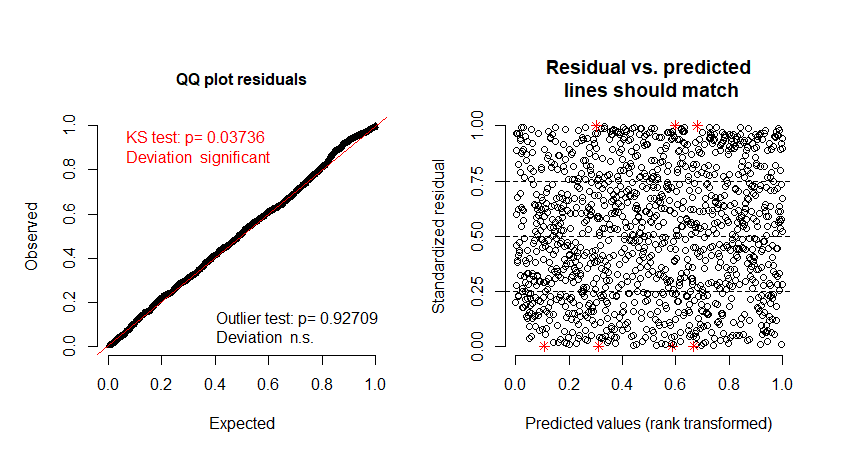


**Figure S10.** Diagnostic plots for DAG-based mixed effects negative binomial models of *S. haematobium* infection intensity (top) and *S. mansoni* infection intensity (bottom)

**References**

1. Hilbe JM. Negative Binomial Regression. 2nd Edition. Cambridge, UK: Cambridge University Press; 2011.

2. Kleinbaum DG, Kleinbaum DG, editors. Applied regression analysis and other multivariable methods. 4th ed. Australia ; Belmont, CA: Brooks/Cole; 2007. 906 p.

3. Gelman A, Hill J. Data analysis using regression and multilevel/hierarchical models [Internet]. Cambridge; New York: Cambridge University Press; 2007 [cited 2020 Feb 3]. Available from: https://doi.org/10.1017/CBO9780511790942
