# Supplementary material for: Land use impacts on parasitic infection: a cross-sectional epidemiological study on the role of irrigated agriculture in schistosome infection in a dammed landscape
Source: Infect Dis Poverty. 2021 Mar 22;10:35. doi: 10.1186/s40249-021-00816-5 (PMC7983278; doi:10.1186/s40249-021-00816-5)
Supplement: Supplementary file 8 — Additional file 8. Distribution of egg counts. [file 40249_2021_816_MOESM8_ESM.docx]

**
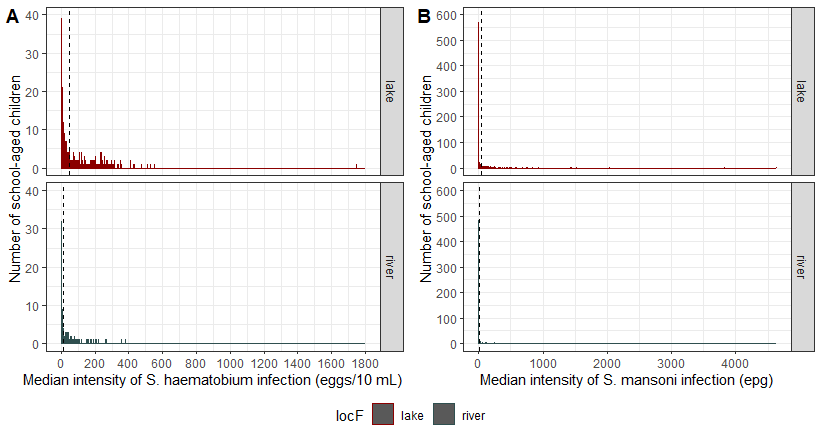
**

**Figure S4.** Distribution of egg counts for (A) *S. haematobium* and (B) *S. mansoni* infections by location strata.
