# Supplementary material for: Land use impacts on parasitic infection: a cross-sectional epidemiological study on the role of irrigated agriculture in schistosome infection in a dammed landscape
Source: Infect Dis Poverty. 2021 Mar 22;10:35. doi: 10.1186/s40249-021-00816-5 (PMC7983278; doi:10.1186/s40249-021-00816-5)
Supplement: Supplementary file 9 — Additional file 9. Sensitivity analysis. [file 40249_2021_816_MOESM9_ESM.docx]

**Sensitivity analysis**

We assessed the sensitivity of our results to a variety of secondary outcome variables (Table S6). Secondary outcome variables included: (1) categorical intensity of *S. haematobium* infection, (2) categorical intensity of *S. mansoni* infection, (3) presence of co-infection with both *S. haematobium* and *S. mansoni* and (4) number of schistosome species present in an individual (an ordinal variable taking on values 0, 1 or 2).

| **Table S6.** Frequencies (%) of secondary outcome variables for *S. haematobium* (Sh) and *S. mansoni* (Sm) infection in the entire study population and across location strata | | | | | |
| --- | --- | --- | --- | --- | --- |
|  | **N** | **Level** | **Overall** | **River** | **Lake** |
| *Sh* infection intensity | 1232 | None | 427 (34.7) | 301 (56.9) | 126 (17.9) |
| (eggs per 10 mL urine) |  | Low (1-49) | 580 (47.1) | 191 (36.1) | 389 (55.3) |
|  |  | High (50+) | 225 (18.3) | 37 (7.0) | 188 (26.7) |
| *Sm* infection intensity | 1222 | None | 1014 (83.0) | 459 (87.1) | 555 (79.9) |
| (eggs per gram feces) |  | Low (1-99) | 131 (10.7) | 47 (8.9) | 84 (12.1) |
|  |  | Moderate (100-399) | 54 (4.4) | 16 (3.0) | 38 (5.5) |
|  |  | High (400+) | 23 (1.9) | 5 (0.9) | 18 (2.6) |
| Co-infection presence | 1060 | No | 1060 (86.4) | 477 (90.2) | 583 (83.5) |
|  |  | Yes | 167 (13.6) | 52 (9.8) | 115 (16.5) |
| Number of infections | 1060 | 0 | 381 (31.3) | 283 (53.7) | 98 (14.2) |
|  |  | 1 | 671 (55.0) | 192 (36.4) | 479 (69.2) |
|  |  | 2 | 167 (13.7) | 52 (9.9) | 115 (16.6) |

Cut points for infection intensity categories were determined by guidelines outlined by the World Health Organization (1). Urogenital *S. haematobium* infection intensity was classified into three categories: (1) no infection (zero eggs per 10 mL urine), (2) low intensity (1-49 eggs per 10 mL urine) and (3) high intensity (50+ eggs per mL urine). Intestinal *S. mansoni* infection intensity was classified into four categories: (1) no infection (zero eggs per gram feces), (2) low intensity (1-99 eggs per gram feces), moderate intensity (100-399 eggs per gram feces) and high intensity (400+ eggs per gram feces). We also examined presence/absence of *S. haematobium*-*S. mansoni* co-infection and the number of infections (e.g. 0, 1 or 2) accumulated. The sample sizes for *S. haematobium* (n = 1232) and *S. mansoni*-specific (n = 1222) models remained the same as the models reported in the main text. Models using multi-infection metrics as the outcome variable (e.g. coinfection and number of infections) were fit with fewer observations (n = 1060) with data on both infection types.

| **Table S7.** Frequencies (%) of secondary measures used to determine a household’s exposure to irrigated agriculture for entire study population and location strata | | | | | |
| --- | --- | --- | --- | --- | --- |
|  | **Level** | **Overall** | | **River** | **Lake** |
| Binary irrigated area  [n (%)] | No | 657 (47.9) | | 288 (50.7) | 369 (45.9) |
|  | Yes | 715 (52.1) | | 280 (49.3) | 435 (54.1) |
| Irrigated area categories  [n (%)] | None | 657 (47.9) | | 288 (50.7) | 369 (45.9) |
|  | > 1 ha | 388 (28.3) | | 178 (31.3) | 210 (26.1) |
|  | 1 – 5 ha | 281 (20.5) | | 89 (15.7) | 192 (23.9) |
|  | < 5 ha | 46 (3.4) | | 13 (2.3) | 33 (4.1) |
| Monocrop area [mean (sd)] |  | 0.71 (1.98) | | 0.86 (2.79) | 0.60 (1.09) |
| Garden area [mean (sd)] |  | 0.30 (0.91) | | 0.09 (0.31) | 0.45 (1.14) |
| Rice area [mean (sd)] |  | 0.32 (1.78) | 0.75 (2.71) | | 0.02 (0.11) |

Secondary exposure variables were also considered in our sensitivity analysis (Table S7). These included binary and categorical formulations of total irrigated area as well as areas of specific types of irrigated land (Table S7). Thresholds for irrigated area categories were based on field observations of small, medium and large areas. Small fields were considered to be less than 1 hectare, while medium fields ranged from 1 to 5 hectares and large fields were greater than 5 hectares. Different types of irrigated area included (1) irrigated monocrops, reflecting the cultivation of a single cash crop (e.g. rice, manioc or onion) on a given field, (2) market gardens, which are typically small areas, often found on the shore of the river or lake, where several vegetable crops are grown for consumption and sale and (3) rice fields, which are the most irrigation-intensive crops grown in the area, relying on the irrigation infrastructure developed immediately upstream of the Diama dam following its construction. As a result, rice fields are predominately located in river villages (Table S7).

The effect of irrigated area on secondary outcomes is largely consistent with the findings reported in the main text, though estimates for *S. haematobium* intensity are now larger and, in some cases, significant. We used a variety of methods to fit models with the multinomial infection intensity categories outcome while still accounting for the nested structure of the data. In all cases, we compare the categories of low, medium and high intensity infection to the reference category of no infection (Table S8). These methods included (1) fitting separate mixed effects logistic regression models for each level comparison, (2) fitting a multinomial regression with village fixed effects and (3) fitting generalized estimating equations (GEE). GEE models were fit using the *multgee* package (version 1.6.0) in R (2,3). The results of all model are reported in Table S8, with the exception of the GEE model for *S. mansoni* intensity, which could not be fit even when specifying initial values.

For *S. haematobium* intensity, we find significant increases in the odds of both low and high intensity infection (Table S8). The effect sizes are generally larger than what we estimated from the mixed effects negative binomial regression and several sets of confidence intervals remain above the null value of 1. For *S. mansoni*, our conclusion about the relationship between irrigated area and infection intensity remains unchanged (Table S2). Point estimates all indicate an increase in infection with irrigated area but confidence intervals remain wide enough to preclude firm conclusions (Table S8).

| **Table S8.** Sensitivity analysis of the effect of irrigated area on secondary outcomes of schistosome infection using multinomial logistic regression. | | | |
| --- | --- | --- | --- |
| **Intensity categories^a^** | **Level** | **Odds Ratio** | **95% CI** |
| Separate logits with nested random intercepts |  |  |  |
| *S. haematobium* | None | REF | REF |
| (eggs per 10 mL urine) | Low (1-49) | 1.16 | 1.03, 1.31 |
|  | High (50+) | 1.11 | 0.98, 1.25 |
| *S. mansoni* | None | REF | REF |
| (eggs per gram feces) | Low (1-99) | 1.01 | 0.92, 1.11 |
|  | Medium/High (100+) | 1.13 | 0.86, 1.48 |
| Multinomial regression with village fixed effects |  |  |  |
| *S. haematobium* | None | REF | REF |
| (eggs per 10 mL urine) | Low (1-49) | 1.13 | 1.02, 1.28 |
|  | High (50+) | 1.16 | 1.03, 1.31 |
| *S. mansoni* | None | REF | REF |
| (eggs per gram feces) | Low (1-99) | 1.00 | 0.90, 1.12 |
|  | Medium (100-399) | 1.05 | 0.89, 1.24 |
|  | High (400+) | 1.12 | 0.86, 1.45 |
| Generalized estimating equations |  |  |  |
| *S. haematobium* | None | REF | REF |
| (eggs per 10 mL urine) | Low (1-49) | 0.12 | 0.10, 0.17 |
|  | High (50+) | 1.52 | 1.17, 2.00 |
| Number of infections |  |  |  |
|  | None | REF | REF |
|  | One | 1.08 | 0.98, 1.18 |
|  | Two | 1.09 | 0.98, 1.20 |
| ^a^ Intensity categories defined by World Health Organization guidelines | | | |

We also examine the effect of different formulations of irrigated area on the presence of schistosome infection (Table S9). For both *S. haematobium* and *S. mansoni,* we see point estimates for the odds of infection increase for medium (1 – 5 ha) and large (< 5 ha) compared to no irrigated area. These effect estimates are larger for *S. haematobium* infections (OR = 1.25 for medium fields; OR = 3.18 for large fields; Table S3) compared to those for *S. mansoni* infection (OR = 1.02 for medium fields; OR = 1.25 for large fields; Table S9). Only one of these estimates, the effect of large fields on *S. haematobium* presence, has a confidence interval completely above the null (95% CI 1.12, 9.00; Table S3). From this, we conclude that the odds of *S. haematobium* infection in school-aged children living in households cultivating large (< 5 ha) fields is three times that of school-aged children living in households that cultivate no land.

| **Table S9.** Sensitivity analysis of the effect of secondary forms of irrigated area on *S. haematobium* and *S. mansoni* infection presence | | | |
| --- | --- | --- | --- |
|  | **Level** | **Odds Ratio** | **95% CI** |
| Irrigated area categories |  |  |  |
| *S. haematobium* | None | REF | REF |
|  | > 1 ha | 0.93 | 0.65, 1.33 |
|  | 1 – 5 ha | 1.25 | 0.78, 2.00 |
|  | < 5 ha | 3.18 | 1.12, 9.00 |
| *S. mansoni* | None | REF | REF |
|  | > 1 ha | 0.94 | 0.59, 1.49 |
|  | 1 – 5 ha | 1.02 | 0.57, 1.80 |
|  | < 5 ha | 1.25 | 0.48, 3.23 |
| Presence of irrigated area |  |  |  |
| *S. haematobium* | Yes | 1.04 | 0.73, 1.46 |
| *S. mansoni* | Yes | 0.97 | 0.63, 1.49 |
| Area of irrigated monocrops |  |  |  |
| *S. haematobium* |  | 1.14 | 1.01, 1.29 |
| *S. mansoni* |  | 0.98 | 0.87, 1.11 |
| Area of market gardens |  |  |  |
| *S. haematobium* |  | 1.11 | 0.91, 1.36 |
| *S. mansoni* |  | 1.12 | 0.94, 1.34 |
| Area of rice cultivation |  |  |  |
| *S. haematobium* |  | 1.07 | 0.95, 1.22 |
| *S. mansoni* |  | 0.96 | 0.79, 1.16 |

Most of the remaining estimates of the effects of different formulations of irrigated area *S. haematobium* infection presence indicate non-significant increases in the odds of infection with irrigated area (Table S9). The size of these estimated effects ranges from a 4% increase in the odds of infection for school-aged children living in households that cultivate any land compared to those living in households that cultivate no land to a 14% percent increase in the odds of infection with each additional hectare of land under irrigated monocrops (Table S9). This estimate of the effect of land area under irrigated monocrops is the only other (beside large areas on *S. haematobium* presence) whose entire confidence interval is above the null value of 1 (Table S9).

With one exception, additional estimates of effect of irrigated area represent slight decreases (2-4%) in the odds of *S. mansoni* infection (Table S9). The one exception is a 12% increase in the odds of infection with each hectare of irrigated land under market gardens (Table S9). However, all estimates of the effect of irrigated area on *S. mansoni* presence have confidence intervals that span the null value (Table S9).

Finally, we examine the effect of different formulations of irrigated area on the intensity of schistosome infection (Table S10). In most of these cases, wide confidence intervals still preclude firm conclusions. However, the intensity of *S. mansoni* infections appears to significantly decrease in school-aged children whose households cultivate small fields (> 1 ha) compared to those in households cultivating no land (RR = 0.27, 95% CI 0.10, 0.78; Table S10).

| **Table S10.** Sensitivity analysis of the effect of secondary formulations of irrigated area on schistosome infection intensity | | | |
| --- | --- | --- | --- |
|  | **Level** | **Rate Ratio** | **95% CI** |
| Irrigated area categories |  |  |  |
| Sh intensity | None | REF | REF |
|  | > 1 ha | 1.05 | 0.69, 1.57 |
|  | 1 – 5 ha | 0.79 | 0.47, 1.36 |
|  | < 5 ha | 1.20 | 0.42, 3.39 |
| Sm intensity^a^ | None | REF | REF |
|  | > 1 ha | 0.27 | 0.10, 0.78 |
|  | 1 – 5 ha | 1.34 | 0.30, 5.92 |
|  | < 5 ha | 1.87 | 0.20, 17.52 |
| Presence of irrigated area |  |  |  |
| Sh intensity | Yes | 0.98 | 0.67, 1.43 |
| Sm intensity^a^ | Yes | 0.47 | 0.17, 1.28 |
| Area of irrigated mono crops |  |  |  |
| Sh intensity |  | 1.06 | 0.98, 1.14 |
| Sm intensity^a^ |  | 0.99 | 0.77, 1.28 |
| Area of market gardens |  |  |  |
| Sh intensity |  | 1.00 | 0.83, 1.22 |
| Sm intensity^a^ |  | 1.17 | 0.86, 1.60 |
| Area of rice cultivation |  |  |  |
| Sh intensity |  | 1.07 | 0.98, 1.16 |
| Sm intensity^a^ |  | 1.06 | 0.76, 1.46 |
| ^a^All models of Sm intensity fit with village random intercepts due to lack of convergence with random intercepts of households nested in villages | | | |

**References**

1. Montresor A, Crompton DWT, Hall A, Bundy D a. P, Savioli L, Unit WHOD of C of TDS and IP. Guidelines for the evaluation of soil-transmitted helminthiasis and schistosomiasis at community level : a guide for managers of control programmes. 1998 [cited 2020 Jan 27]; Available from: https://apps.who.int/iris/handle/10665/63821

2. Nooraee N, Molenberghs G, van den Heuvel ER. GEE for longitudinal ordinal data: Comparing R-geepack, R-multgee, R-repolr, SAS-GENMOD, SPSS-GENLIN. Computational Statistics & Data Analysis [Internet]. 2014 Sep [cited 2020 Mar 15];77:70–83. Available from: https://linkinghub.elsevier.com/retrieve/pii/S0167947314000863

3. Touloumis A. R Package multgee: A Generalized Estimating Equations Solver for Multinomial Responses. J Stat Soft [Internet]. 2015 [cited 2020 Apr 8];64(8). Available from: http://www.jstatsoft.org/v64/i08/
