# Supplementary material for: Land use impacts on parasitic infection: a cross-sectional epidemiological study on the role of irrigated agriculture in schistosome infection in a dammed landscape
Source: Infect Dis Poverty. 2021 Mar 22;10:35. doi: 10.1186/s40249-021-00816-5 (PMC7983278; doi:10.1186/s40249-021-00816-5)
Supplement: Supplementary file 10 — Additional file 10. Supplementary data for alternative models. [file 40249_2021_816_MOESM10_ESM.docx]

**Supplementary data for non-DAG-based models**

**Table S14.** Regression output for alternative crude, adjusted and mixed effects logistic regression models of *S. haematobium* (Sh_presence) and *S. mansoni* (Sm_presence) infection presence.

|  | *Dependent variable:* | | | | | |
| --- | --- | --- | --- | --- | --- | --- |
|  |  | | | | | |
|  | Sh_presence | | | Sm_presence | | |
|  | *logistic* | | *generalized linear* | *logistic* | | *generalized linear* |
|  |  | | *mixed-effects* |  | | *mixed-effects* |
|  | (1) | (2) | (3) | (4) | (5) | (6) |
|  | | | | | | |
| Constant | 0.51^***^ | 2.08^***^ | 2.08^***^ | -1.64^***^ | -2.39^***^ | -1.82^**^ |
|  | (0.07) | (0.47) | (0.54) | (0.08) | (0.63) | (0.72) |
|  |  |  |  |  |  |  |
| Irrigated area | 0.15^***^ | 0.10^*^ | 0.11^**^ | 0.06^*^ | -0.01 | 0.01 |
|  | (0.04) | (0.05) | (0.05) | (0.03) | (0.04) | (0.05) |
|  |  |  |  |  |  |  |
| pumpYN |  | -0.25 | -0.40^*^ |  | -0.08 | -0.20 |
|  |  | (0.21) | (0.22) |  | (0.22) | (0.25) |
|  |  |  |  |  |  |  |
| Location (river) |  | -1.48^***^ | -1.70^***^ |  | 0.36 | 0.40 |
|  |  | (0.22) | (0.36) |  | (0.28) | (0.48) |
|  |  |  |  |  |  |  |
| Sex (female) |  | -0.38^***^ | -0.40^***^ |  | -0.26 | -0.26 |
|  |  | (0.14) | (0.15) |  | (0.17) | (0.17) |
|  |  |  |  |  |  |  |
| Age |  | 0.12 | 0.13 |  | 0.01 | 0.07 |
|  |  | (0.08) | (0.08) |  | (0.09) | (0.09) |
|  |  |  |  |  |  |  |
| Head, 1-7 yrs school |  | 0.17 | 0.16 |  | -15.69 | -15.22 |
|  |  | (0.45) | (0.46) |  | (556.32) | (757.64) |
|  |  |  |  |  |  |  |
| Head, 7+ yrs school |  | -0.19 | -0.15 |  | -0.24 | -0.37 |
|  |  | (0.19) | (0.20) |  | (0.25) | (0.26) |
|  |  |  |  |  |  |  |
| # wives (1) |  | 0.06 | 0.01 |  | -0.10 | -0.13 |
|  |  | (0.25) | (0.25) |  | (0.29) | (0.30) |
|  |  |  |  |  |  |  |
| # wives (2+) |  | -0.17 | -0.16 |  | 0.05 | -0.03 |
|  |  | (0.27) | (0.28) |  | (0.31) | (0.32) |
|  |  |  |  |  |  |  |
| Ethnicity (other) |  | 0.03 | 0.11 |  | 0.75 | 0.28 |
|  |  | (0.45) | (0.48) |  | (0.77) | (0.80) |
|  |  |  |  |  |  |  |
| Ethnicity (Pulaar) |  | 0.08 | 0.31 |  | 0.51 | -0.10 |
|  |  | (0.31) | (0.33) |  | (0.53) | (0.57) |
|  |  |  |  |  |  |  |
| Ethnicity (Wolof) |  | 0.32 | 0.44 |  | 1.55^***^ | 0.95^*^ |
|  |  | (0.29) | (0.31) |  | (0.47) | (0.51) |
|  |  |  |  |  |  |  |
| # fishermen |  | 0.10 | 0.08 |  | -0.02 | -0.03 |
|  |  | (0.09) | (0.10) |  | (0.10) | (0.10) |
|  |  |  |  |  |  |  |
| Kids agricultural tasks |  | -0.08 | -0.13 |  | -0.02 | 0.54 |
|  |  | (0.39) | (0.41) |  | (0.50) | (0.52) |
|  |  |  |  |  |  |  |
| Number of WP |  | 0.20^**^ | 0.18 |  | 0.19^*^ | 0.20 |
|  |  | (0.10) | (0.11) |  | (0.10) | (0.12) |
|  |  |  |  |  |  |  |
| # Agricultural WP |  | -0.04 | 0.01 |  | -0.27^***^ | -0.22^**^ |
|  |  | (0.08) | (0.08) |  | (0.09) | (0.10) |
|  |  |  |  |  |  |  |
| Asset quintile 2 |  | -0.12 | 0.05 |  | -0.43 | -0.50 |
|  |  | (0.27) | (0.28) |  | (0.32) | (0.33) |
|  |  |  |  |  |  |  |
| Asset quintile 3 |  | -0.41 | -0.32 |  | -0.47 | -0.64^*^ |
|  |  | (0.26) | (0.27) |  | (0.31) | (0.33) |
|  |  |  |  |  |  |  |
| Asset quintile 4 |  | -0.16 | -0.002 |  | -0.22 | -0.27 |
|  |  | (0.26) | (0.26) |  | (0.29) | (0.30) |
|  |  |  |  |  |  |  |
| Asset quintile 5 |  | -0.36 | -0.10 |  | -0.49^*^ | -0.49 |
|  |  | (0.26) | (0.27) |  | (0.30) | (0.32) |
|  |  |  |  |  |  |  |
| Distance to water point |  | -0.49^***^ | -0.47^***^ |  | -0.23 | -0.30^*^ |
|  |  | (0.09) | (0.10) |  | (0.14) | (0.17) |
|  |  |  |  |  |  |  |
| Distance to market |  | 0.53^***^ | 0.51^***^ |  | -0.76^***^ | -0.75^***^ |
|  |  | (0.09) | (0.16) |  | (0.10) | (0.21) |
|  |  |  |  |  |  |  |
| Village irrigated area |  | 0.0001 | -0.001 |  | 0.0004 | -0.0003 |
|  |  | (0.001) | (0.002) |  | (0.001) | (0.002) |
|  |  |  |  |  |  |  |
|  | | | | | | |
| Observations | 1,232 | 1,232 | 1,232 | 1,222 | 1,222 | 1,222 |
| Log Likelihood | -788.05 | -610.16 | -600.89 | -555.76 | -475.92 | -459.64 |
| Akaike Inf. Crit. | 1,580.10 | 1,268.33 | 1,253.77 | 1,115.52 | 999.85 | 971.28 |
| Bayesian Inf. Crit. |  |  | 1,386.80 |  |  | 1,104.09 |
|  | | | | | | |
| *Note:* | ^*^p^**^p^***^p<0.01 | | | | | |


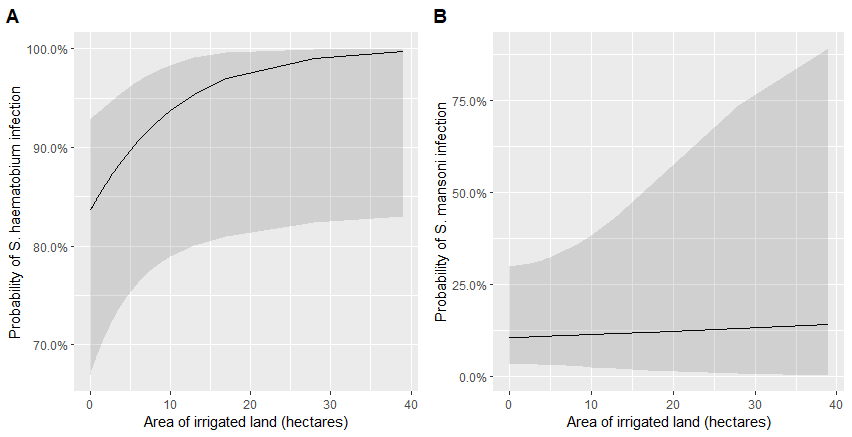


**Figure S11.** Prediction plots for mixed effects logistic regression models for the alternative (e.g. non-DAG-based) specification of the model, where (A) probability of *S.* haematobium infection and (B) probability of *S. mansoni* infection across observed values of irrigated land area.


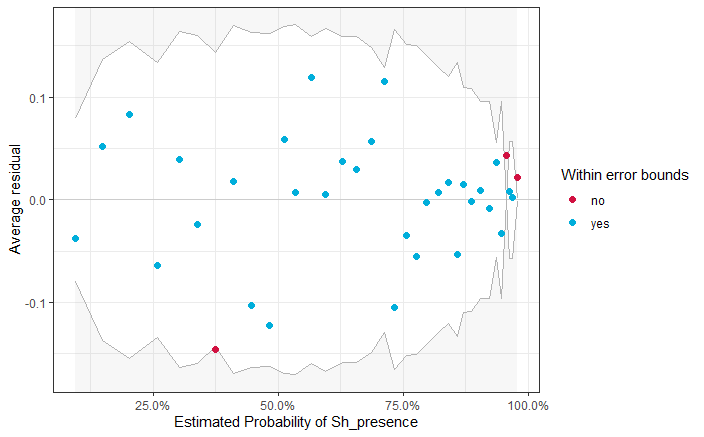

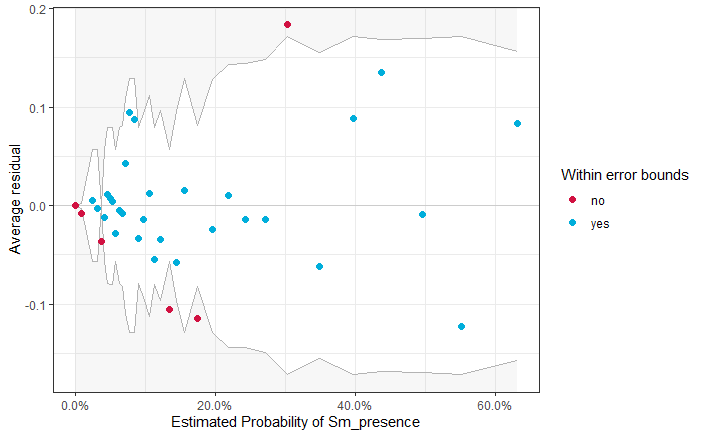


**Figure S12.** Binned residual plots for non-DAG-based mixed effects logistic regression models of schistosome infection presence. Left, 91% of binned residuals of *S. haematobium* presence fall within error bounds. Right, 83% of binned residuals of *S. mansoni* presence fall within error bounds.


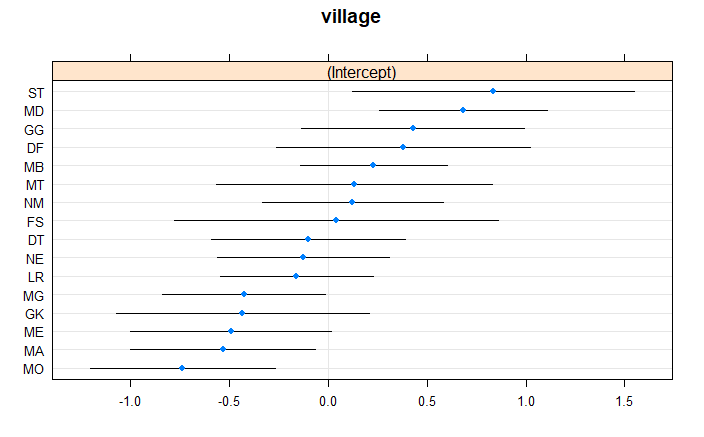

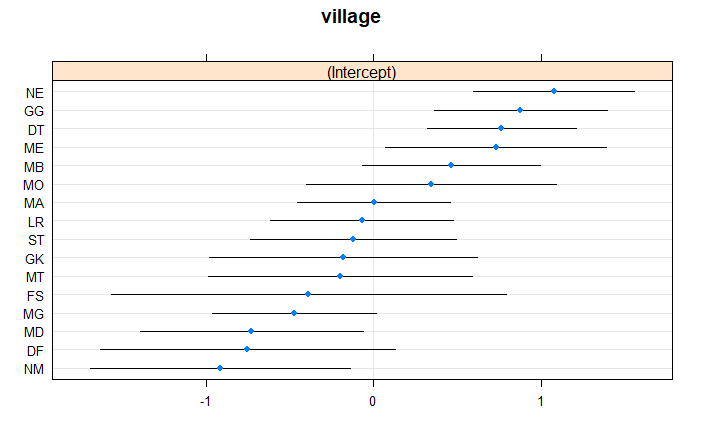


**Figure S13.** Point estimate and confidence intervals for village random intercepts in non-DAG-based mixed effects logistic regression models of *S. haematobium* presence (left) and *S. mansoni* presence (right)


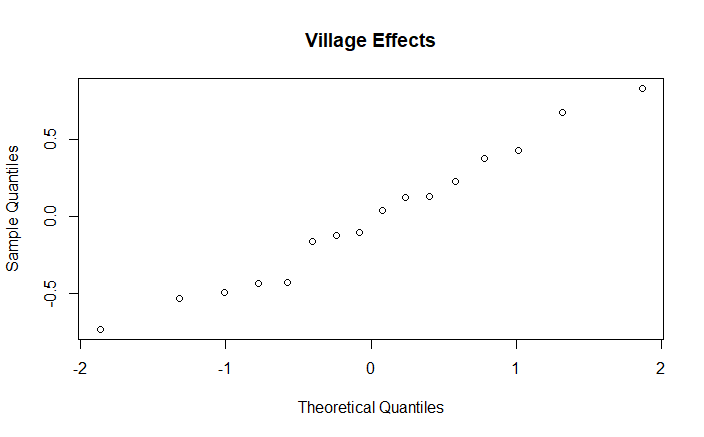

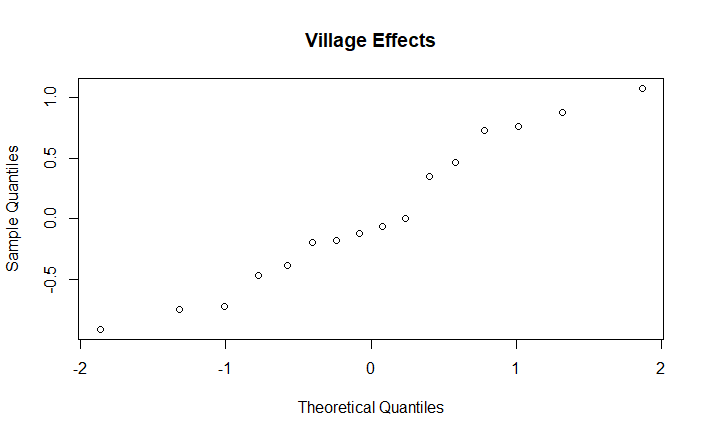


**Figure S14.** Q-Q plot of village random effects for non-DAG-based mixed effects logistic regression models of *S. haematobium* presence (left) and *S. mansoni* presence (right)

**Table S15.** Regression output for alternative of crude, adjusted and mixed effects negative binomial models of *S. haematobium* (Sh_median) and *S. mansoni* (Sm_median) infection intensity.

|  | | | | | | |
| --- | --- | --- | --- | --- | --- | --- |
|  | *Dependent variable:* | | | | | |
|  |  | | | | | |
|  | Sh_median | | | Sm_median | | |
|  | (1) | (2) | (3) | (4) | (5) | (6) |
|  | | | | | | |
| Constant | 3.55^***^ | 4.01^***^ | 3.97^***^ | 3.49^***^ | 3.42*^*^ | 3.98 |
|  | (0.07) | (0.43) | (0.64) | (0.22) | (1.52) | (2.26) |
|  |  |  |  |  |  |  |
| Irrigated area | 0.01 | 0.04 | 0.05 | 0.02 | 0.03 | 0.06 |
|  | (0.03) | (0.03) | (0.04) | (0.09) | (0.10) | (0.13) |
|  |  |  |  |  |  |  |
| pumpYN |  | 0.14 | -0.20 |  | -0.48 | -1.32 |
|  |  | (0.19) | (0.24) |  | (0.56) | (0.76) |
|  |  |  |  |  |  |  |
| Location (river) |  | -1.24^***^ | -2.28^***^ |  | 0.98 | 0.49 |
|  |  | (0.21) | (0.50) |  | (0.67) | (1.32) |
|  |  |  |  |  |  |  |
| Sex (female) |  | -0.08 | -0.21 |  | -0.99* | -0.93 |
|  |  | (0.14) | (0.15) |  | (0.43) | (0.52) |
|  |  |  |  |  |  |  |
| Age |  | -0.31^***^ | -0.22 |  | -0.99 | 0.19 |
|  |  | (0.07) | (0.08) |  | (0.43) | (0.25) |
|  |  |  |  |  |  |  |
| Head, 1-7 yrs school |  | 0.28 | 0.18 |  | -21.90 | -23.66 |
|  |  | (0.37) | (0.47) |  | (1926.91) | (2864.26) |
|  |  |  |  |  |  |  |
| Head, 7+ yrs school |  | -0.76^***^ | -0.43 |  | -1.71** | -2.39** |
|  |  | (0.18) | (0.24) |  | (0.59) | (0.79) |
|  |  |  |  |  |  |  |
| # wives (1) |  | 0.01 | -0.33 |  | -0.65 | -0.82 |
|  |  | (0.26) | (0.32) |  | (0.83) | (0.90) |
|  |  |  |  |  |  |  |
| # wives (2+) |  | 0.04 | -0.23 |  | -1.72 | -2.12* |
|  |  | (0.30) | (0.36) |  | (0.93) | (1.04) |
|  |  |  |  |  |  |  |
| Ethnicity (other) |  | 0.35 | 0.37 |  | -3.67* | -4.52 |
|  |  | (0.49) | (0.61) |  | (1.69) | (2.53) |
|  |  |  |  |  |  |  |
| Ethnicity (Pulaar) |  | -0.18 | 0.03 |  | -1.21 | -3.68* |
|  |  | (0.35) | (0.46) |  | (1.33) | (1.76) |
|  |  |  |  |  |  |  |
| Ethnicity (Wolof) |  | 0.14 | -0.10 |  | 2.54 | 1.37 |
|  |  | (0.31) | (0.40) |  | (1.33) | (1.67) |
|  |  |  |  |  |  |  |
| # fishermen |  | 0.02 | -0.09 |  | 0.00 | -0.33 |
|  |  | (0.09) | (0.10) |  | (0.33) | (0.39) |
|  |  |  |  |  |  |  |
| Kids agricultural tasks |  | -0.48 | -0.44 |  | 1.13 | 2.60 |
|  |  | (0.41) | (0.57) |  | (1.67) | (1.82) |
|  |  |  |  |  |  |  |
| Number of WP |  | -0.04 | 0.01 |  | -0.26 | 0.47 |
|  |  | (0.30) | (0.12) |  | (0.25) | (0.36) |
|  |  |  |  |  |  |  |
| # Agricultural WP |  | -0.09 | -0.05 |  | -0.42^*^ | -0.38 |
|  |  | (0.07) | (0.09) |  | (0.21) | (0.28) |
|  |  |  |  |  |  |  |
| Asset quintile 2 |  | -0.29 | -0.19 |  | 0.07 | -0.53 |
|  |  | (0.25) | (0.31) |  | (0.90) | (1.08) |
|  |  |  |  |  |  |  |
| Asset quintile 3 |  | -0.19 | -0.21 |  | -1.52 | -2.35^*^ |
|  |  | (0.24) | (0.30) |  | (0.92) | (0.97) |
|  |  |  |  |  |  |  |
| Asset quintile 4 |  | -0.23 | 0.06 |  | -0.23 | -0.54 |
|  |  | (0.24) | (0.30) |  | (0.82) | (0.93) |
|  |  |  |  |  |  |  |
| Asset quintile 5 |  | -0.57* | -0.19 |  | -0.78 | -0.68 |
|  |  | (0.25) | (0.32) |  | (0.84) | (0.97) |
|  |  |  |  |  |  |  |
| Distance to water point |  | -0.18 | -0.54^***^ |  | -0.36 | -0.30 |
|  |  | (0.09) | (0.12) |  | (0.32) | (0.38) |
|  |  |  |  |  |  |  |
| Distance to market |  | 0.25^***^ | 0.45^*^ |  | -2.25^***^ | -2.53^***^ |
|  |  | (0.08) | (0.22) |  | (0.25) | (0.61) |
|  |  |  |  |  |  |  |
| Village irrigated area |  | 0.00 | 0.00 |  | -0.00 | -0.01 |
|  |  | (0.00) | (0.00) |  | (0.00) | (0.01) |
|  |  |  |  |  |  |  |
|  | | | | | | |
| Observations | 1,232 | 1,232 | 1,232 | 1,222 | 1,222 | 1,222 |
| Log Likelihood | -4,320.36 | -4,235.98 | -4,157.11 | -1,625.62 | -1,559.85 | -1,547.586 |
| Akaike Inf. Crit. | 8,646.71 | 8,521.96 | 8,368.23 | 3,257.62 | 3,169.70 | 3,149.17 |
|  | | | | | | |
| *Note:* | ^*^p^**^p^***^p<0.01 | | | | | |


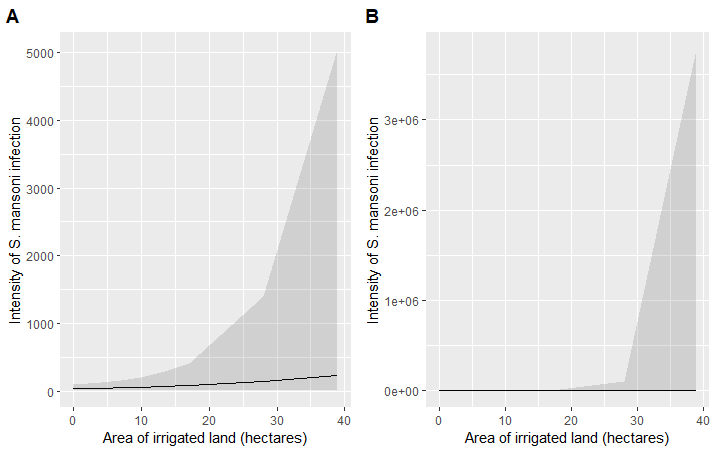


**Figure S15.** Prediction plots for non-DAG-based mixed effects negative binomial models of (A) intensity of *S. haematobium* infection and (B) intensity of *S. mansoni* infection across observed values of irrigated land area


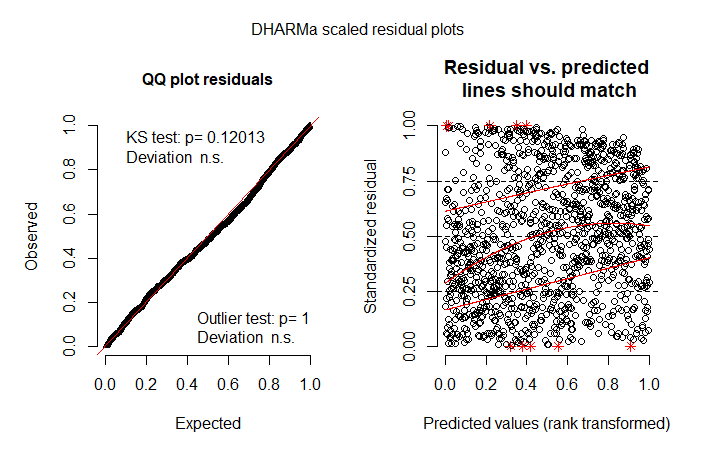


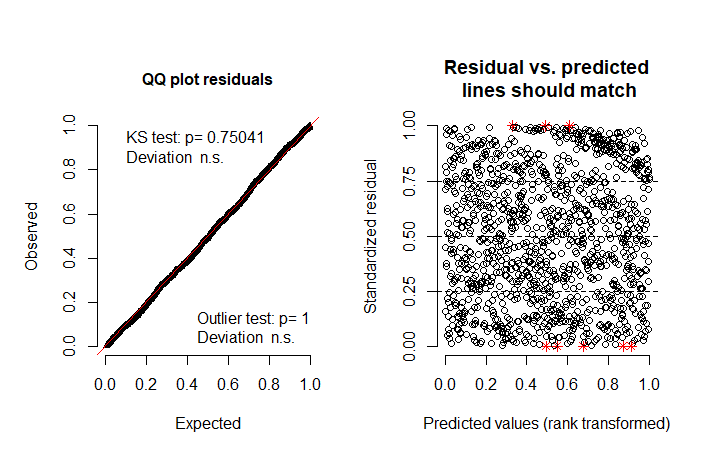


**Figure S16.** Diagnostic plots for non-DAG-based mixed effects negative binomial models of *S. haematobium* infection intensity (top) and *S. mansoni* infection intensity (bottom)
